# Focus on Extracellular Vesicles: Introducing the Next Small Big Thing

**DOI:** 10.3390/ijms17020170

**Published:** 2016-02-06

**Authors:** Hina Kalra, Gregor P. C. Drummen, Suresh Mathivanan

**Affiliations:** 1 Department of Biochemistry and Genetics, La Trobe Institute for Molecular Science, La Trobe University, Melbourne, VIC 3086, Australia; hkalra@students.latrobe.edu.au; 2 Cellular Stress and Ageing Program, Bionanoscience and Bio-Imaging Program, Bio&Nano-Solutions, D-33647 Bielefeld, Germany

**Keywords:** exosome, ectosome, microvesicle, apoptotic body, extracellular vesicle, molecular composition, signal transduction, biogenesis, isolation

## Abstract

Intercellular communication was long thought to be regulated exclusively through direct contact between cells or via release of soluble molecules that transmit the signal by binding to a suitable receptor on the target cell, and/or via uptake into that cell. With the discovery of small secreted vesicular structures that contain complex cargo, both in their lumen and the lipid membrane that surrounds them, a new frontier of signal transduction was discovered. These “extracellular vesicles” (EV) were initially thought to be garbage bags through which the cell ejected its waste. Whilst this is a major function of one type of EV, *i.e.*, apoptotic bodies, many EVs have intricate functions in intercellular communication and compound exchange; although their physiological roles are still ill-defined. Additionally, it is now becoming increasingly clear that EVs mediate disease progression and therefore studying EVs has ignited significant interests among researchers from various fields of life sciences. Consequently, the research effort into the pathogenic roles of EVs is significantly higher even though their protective roles are not well established. The “Focus on extracellular vesicles” series of reviews highlights the current state of the art regarding various topics in EV research, whilst this review serves as an introductory overview of EVs, their biogenesis and molecular composition.

## 1. Introduction

**Figure 1:**
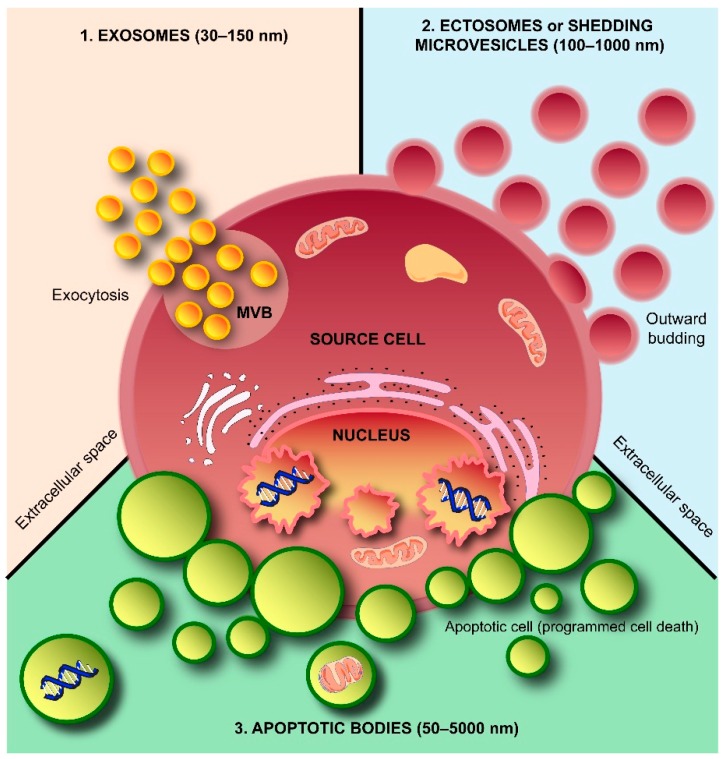
Schematic representation of subtypes of extracellular vesicles (EVs) released by a cell. Three subtypes of EVs, namely exosomes, shedding microvesicles or ectosomes and apoptotic bodies, are known to be secreted by a cell into the extracellular space. Exosomes are released by exocytosis, whereas shedding microvesicles or ectosomes are secreted by outward budding of the plasma membrane. Apoptotic bodies are released by dying cells during the later stages of apoptosis so that cell debris can easily be eliminated by neighboring and immune system cells. MVB: multivesicular body.

Intercellular communication is mostly thought to be mediated by direct cellular interaction or through the secretion of soluble factors [1]. Recently, extracellular vesicles (EVs) are proposed as a novel mode of intercellular communication for both short and longer-range signaling events [2,3,4]. EVs (Figure 1) carry a rich cargo of DNA, RNA, proteins, lipids and metabolites reflective of their cellular origin and are released into the extracellular space by multiple cell types during both physiological and pathological conditions [4,5]. Whilst the role of EVs in normal physiology is poorly understood, their role in pathological conditions is relatively well characterized [6]. EVs have been isolated from many biological fluids, including blood, milk, saliva, malignant ascites, amniotic fluid and urine [7,8,9]. Though the presence of proteins in EVs was reported alongside the discovery of EVs [10], the existence of RNA in EVs was only demonstrated during the past decade.

In 2007, Valadi *et al.* were the first to confirm the presence of RNA inside EVs and also showed that mRNA inside EVs could be translated into proteins *in vitro* [11]*.* Interestingly, the secretion of EVs is conserved in multiple species and thus EVs from one species have the potential to regulate cellular processes in another species, either by inducing benefit (e.g., cow milk exosomes in humans—at least infants) or mediating disease/infection (e.g., fungal exosomes in plants/humans) [12]. In addition, EVs were shown to carry single-stranded DNA (ssDNA), amplified oncogene sequences, transposable elements and mitochondrial DNA [3,13]. Though the presence of mitochondrial DNA has not been validated by other groups, double stranded DNA (dsDNA) in tumor-derived EVs was also discovered and reported recently by several research groups [14,15,16]. This unparalleled horizontal transfer of multiple gene and protein products among cells was until recently considered impossible because some researchers argued that such transfers violate the cell’s autonomy [17,18,19].

**Figure 2:**
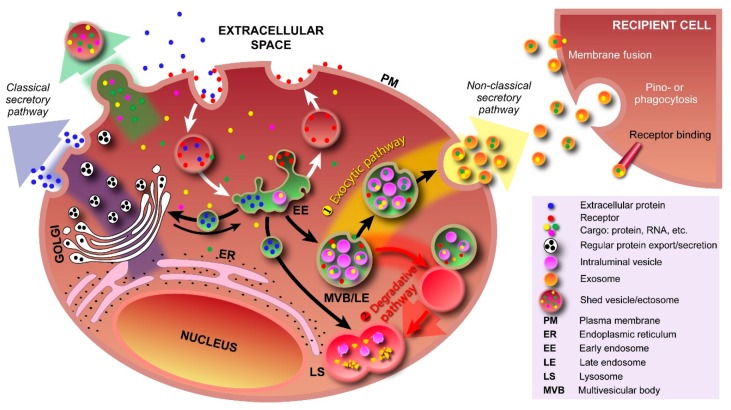
Pathways involving various types of vesicles. In the classical secretory pathway, vesicles with protein cargo, sorted and packed in the Golgi apparatus, transport their cargo to the plasma membrane (PM). By fusing with the PM, both membrane proteins and secretory proteins are effectively transported to their intended destinations. Various types of cargo, e.g., proteins, RNA, can also be transported into the extracellular space via outward PM budding and formation of shedded vesicles (ectosomes). Cargo is taken up by the cell via endocytosis (receptor-mediated and free uptake) and formation of early endosomes. In early endosomes, proteins are either recycled to the PM or sequestered into the intraluminal vesicles (ILV) of MVBs. Formation of exosomes starts with inward budding of the early endosome’s membrane and subsequent formation of MVBs. In the exocytic pathway ①, MVBs fuse with the PM to release their contents (exosomes) into the extracellular space; In the degradative pathway ②, the MVBs are trafficked to lysosomes for enzyme-assisted degradation. This pathway is particularly important for restricting signaling by activated growth factor receptors. Exosomal cargo delivery to the recipient cell can occur through various mechanisms, *i.e.*, direct fusion with the recipient cell’s membrane, pinocytosis/phagocytosis, or ligand–receptor binding.

EVs can broadly be divided into three categories based on the current state of knowledge of their biogenesis. Discrete biogenesis pathways result in subsets of EVs namely: (i) exosomes; (ii) ectosomes or shedding microvesicles (SMVs); and (iii) apoptotic bodies (ABs), as schematically depicted in Figure 1. A common feature in all the three EV subtypes is a lipid bilayer membrane that surrounds a specific cargo of biomolecules, e.g., proteins, RNA, or cellular debris. However, their size and buoyant densities vary significantly [20]; albeit that both size and buoyant density ranges for the various EV subtypes have been heterogeneously reported in the literature. Nonetheless, exosomes are thought to be around 30–150 nm in diameter and have a buoyant density of 1.10–1.14 g/mL. Furthermore, exosomes display cup-like morphology when observed under the transmission electron microscopy [20,21,22]. When discovered more than three decades ago, exosomes were initially thought to be a mechanism of discarding plasma membrane (PM) proteins in maturing reticulocytes [10,23]. These small membranous vesicles are formed by inward budding of endosomal membranes, resulting in the progressive accumulation of intraluminal vesicles (ILVs) within large multivesicular bodies (MVBs) as shown in Figure 2. MVBs can either traffic to lysosomes for degradation (degradative MVBs) or, alternatively, to the PM where, upon fusion with the PM, they release their contents (the ILVs) into the extracellular space (exocytic MVBs). ILVs released into the extracellular space are referred to as “exosomes” (Figure 2). Among the EV subtypes, exosomes have been and are extensively studied [5,20]. While multiple studies have implicated Alix, TSG101, CD63 and CD9 as exosomal markers [20], it is becoming clear that these molecules are enriched in exosomes, but are not markers *per se* as considered previously [24]. In agreement with this, Keerthikumar *et al.* identified enrichment of Alix, TSG101, CD9 and CD63 in exosomes compared to ectosomes [22]. Their study further confirmed that CD81 might distinctly be utilized as an exosomal marker which was further supported by Minciacchi *et al.* [25].

Contrary to exosomes, ectosomes (SMVs) are large vesicles ranging from 100–1000 nm in diameter [26], ubiquitously assembled at and released from the PM through outward protrusion or budding (Figure 2). Ectosomes were first defined by Stein and Luzio when they observed ectocytosis and shedding of PM vesicles in stimulated neutrophils [27]. The rate of ectosome shedding has been observed to be variable between various cell types, but even resting cells shed ectosomes at a low rate. Unlike exosomes, the molecular composition of ectosomes is still largely unknown, but matrix metalloproteinases (MMPs) [28,29,30,31], glycoproteins, e.g., GPIb, GPIIb–IIIa and P-selectin [32,33,34,35], and integrins, e.g., Mac-1, [35,36] seem to be enriched in ectosomes, depending on the cell type. Recent studies also suggest that MMP2 might be utilized as a marker of ectosomes [22,37]. However, ectosomal enriched proteins are largely cell type dependent. For instance, the epithelial cell marker CK18 was enriched in ectosomes [22] and oncosomes [25] secreted by epithelial cells and hence cannot be utilized as markers of ectosomes secreted by fibroblasts. Oncosomes are larger vesicles ranging from 1 to 10 μm in diameter that are thought to follow the biogenesis pathway of ectosomes and are extensively studied by Di Vizio and colleagues [25,38]. Though abundance of large oncosomes in patient plasma and tissue biopsies are shown to be correlated with tumor progression, until now, these large oncosomes are exclusively shown to be released by prostate cancer cells and are poorly characterized in comparison to exosomes [25,39].

Apoptotic bodies (Figure 1) are heterogeneous vesicles that are known to be released from cells undergoing apoptotic cell clearance [40,41] and are thought to be around 50–5000 nm in diameter [20]. Apoptosis or programmed cell death [41], first introduced by Kerr and co-workers in 1972 [42], and the subsequent phagocytic corpse removal are essential during embryonic development, growth, and maintenance of multicellular organisms. Furthermore, apoptosis ensures the selective removal of aged, damaged, infected or aberrant cells from healthy tissues. Essentially, apoptosis is the coordinated dismantling of the cell and cellular debris is packed into ABs. These vesicular structures have external features that trigger phagocytosis; the final step in cell dismantling and recycling of biomolecule building blocks.

The “Focus on extracellular vesicles” series of reviews highlights recent developments in EV research and their role in normal physiology, degenerative and cancerous diseases, and as emerging novel therapeutics [43,44,45,46,47]. The following sections of this introductory review offer a compact overview of various aspects of extracellular vesicles—THE NEXT SMALL BIG THING.

## 2. Exosomes and Colleagues—The Next Small Big Thing

### 2.1. Exosome Biogenesis

**Figure 3:**
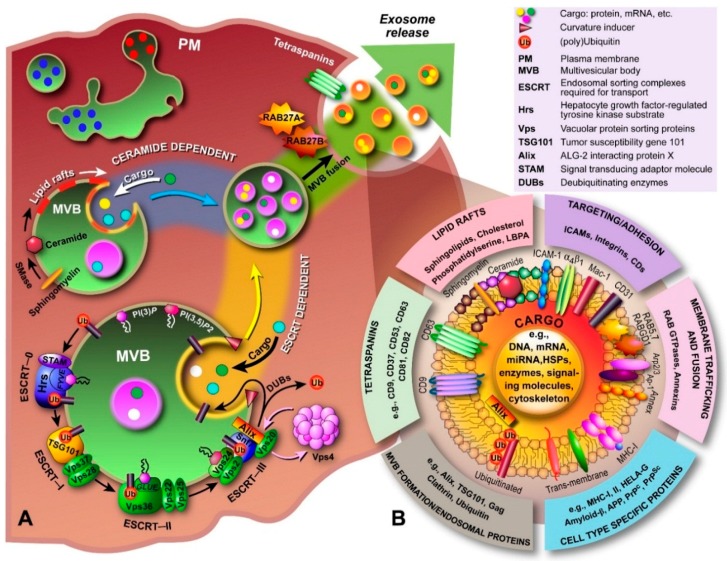
Biogenesis, secretion and composition of exosomes. (**A**) The biogenesis and secretion of exosomes is believed to be mediated via a ceramide and/or ESCRT-dependent pathway. The ceramide-dependent pathway is based on the formation of lipid rafts in which sphingomyelin is converted to ceramide by sphingomyelinases. These ceramide-enriched domains have structural imbalances between monoleaflets causing the membrane to bend inward. In the ESCRT-dependent pathway, components of the ESCRT machinery are sequentially recruited to the endosomal membrane, which starts with Hrs, and bind to phosphatidylinositol-3-phosphate (PI(3)*P*) and the 3,5-bisphosphate (PI(3,5)*P*2) through lipid binding domains (e.g., *FYVE*, *GLUE*), and to the ubiquitinated protein (ESCRT-0). ESCRT-I and -II drive budding of ILVs, during which cargo is transported into the lumen, and ESCRT-III is recruited by Alix to complete budding and drive vesicle scission (spiral formation and pulling). DUBs deubiquitinate the protein and Vps4 recycles the ESCRT machinery. The now formed MVB is transported to the PM and through fusion, the ILVs are released into the extracellular environment and are now called “exosomes”; (**B**) Exosomal luminal cargo predominantly consists of mRNA, miRNA and gDNA fragments, and a myriad of different proteins depending on the cell of origin. Generally, proteins involved in MVB formation, tetraspanins, membrane transport and fusion, transmembrane proteins, cytoskeletal components and proteins of cytosolic origin are part of exosomes. In addition, biomolecules associated with various diseases, including cancer, neurodegenerative diseases, such as Parkinson’s, Alzheimer’s and transmissible spongiform encephalopathies (prion disease), and inflammatory disorders have been identified in exosomes.

The processes that govern the formation of ILVs inside MVBs and the ensuing fusion with the PM to release exosomes into the extracellular space (Figure 2) are incompletely understood. One of the proposed molecular machineries implicated in the biogenesis and secretion of exosomes is the Endosomal Sorting Complex Required for Transport (ESCRT) [48]. In conjunction with a number of accessory proteins, the ESCRT machinery is predominantly involved in binding, sorting, and clustering of ubiquitinylated proteins and receptors. The process of ILV formation starts when the endosomal membrane is reorganized into specialized tetraspanins-enriched microdomains (TEMs), with the involvement of CD9 and CD63, that function to cluster the ILV formation machinery [49]. Tetraspanins are transmembrane proteins that contain four transmembrane domains, N- and C-terminal cytoplasmic tails (<20 residues), and two unequally sized extracellular domains (ED; Short ED < 30; Long ED 76–131 residues) [50]. The ESCRT machinery comprises of ESCRT-0, I, II, and III, which act sequentially to sort ubiquitinylated proteins in the late endosome, as shown schematically in Figure 3. The abundant presence of phosphatidylinositol 3-phosphate (PI(3)*P*) and the ubiquitinated proteins results in binding of Hrs (ESCRT-0 subunit) to PI(3)*P* via its FYVE domain and the ubiquitinated protein. Subsequently, Hrs/STAM recruits ESCRT-I (TSG101 and Vps28) to the endosomal membrane and forms an ESCRT-0/ESCRT-I complex. Next, segregation of ubiquitinylated proteins into microdomains occurs and mobilization of ESCRT-II (Vps22) to the membrane. ESCRT-I and ESCRT-II then initiate reverse budding of nascent ILVs within MVBs and uptake of cytosolic cargo (e.g., RNAs and proteins). Recruitment of ESCRT-III subunits (Alix and Vps2) by ESCRT-II and oligomerization of ESCRT-III subunits inside the neck of the nascent ILVs results in closing of the cargo-containing vesicle and pinching off of the vesicles. How ESCRT-III oligomerization induces membrane curvature has remained elusive. However, recent *in vitro* research by Chiaruttini *et al.* has shown that the major component of ESCRT-III, Snf7 (Figure 3), oligomerizes into spring-like spirals at the lipid membrane surface [51]. The authors observed that elastic expansion of compressed Snf7 spirals induced area differences between endo- and exofacial membrane sides with the consequence that membrane curvature was induced. However, whether an analogous process occurs in ILVs *in vivo* remains to be established. Overall, the components of ESCRT-0, I and II are responsible for sequestering ubiquinated proteins at the endosomal membrane, whereas ESCRT-III contributes towards vesicle closure and detachment of ILVs from the membrane [52,53,54,55]; the accessory proteins, in particular the AAA-ATPase Vps4, are involved in the dissociation and recycling of the ESCRT machinery. Most importantly, ESCRT-III in conjunction with deubiquitinating enzymes, such as HD-PTP, directs deubiquitination of proteins. Alix was recently shown to promote intraluminal budding of vesicles in endosomes upon interaction with syntenin [54]; the cytoplasmic adaptor of syndecan heparan sulphate proteoglycans. Furthermore, interaction of Alix with the ESCRT machinery seems to be driving the accumulation of luminal cargo [56,57].

Besides the ESCRT-dependent pathway, recent research implicates the existence of an ESCRT-independent pathway that involves glycolipoprotein microdomains, *i.e.*, lipid rafts. Indeed, Stuffers *et al.* have shown that MVBs can still be formed in cells depleted of all four ESCRT components, which confirms the presence of an alternate pathway [58]; although some aberrant ILV morphology was observed, whilst the early and late endosomes remained clearly differentiated. Further evidence comes from research on oligodendrocytes in which Trajkovic *et al.* showed that sorting of proteolipid proteins into ILVs is ESCRT independent [59]. In the ESCRT-independent pathway, ILVs and exosome formation are thought to involve the conversion of sphingomyelin to ceramide by sphingomyelinases (Figure 3) [59]. Although this pathway is not fully elucidated, sphingomyelin is shown to be clustered in lipid rafts (enrichment with cholesterol), where it is then converted to ceramide. Ceramide accumulation then induces microdomain coalescence and triggers ILV formation. Although a recent *in vitro* study involving giant unilammellar vesicles questioned this “lipid-only” hypothesis of ILV formation [60], since the authors found that no particular lipid magic bullet was required for ILV formation, several discrepancies between *in vitro* and *in vivo* observations preclude dismissing the ceramide-dependent pathway altogether. Presumably, the two pathways are not clearly separated but occur concomitantly or one becomes dominant in response to the cargo’s physical properties.

Once ILVs are formed in the MVB, trafficking to the cell periphery, subsequent fusion with the PM, and exosome release into the extracellular space all require coordinated and multilevel changes in cytoskeletal-PM interactions, local enzymatic degradation, and the activation of the fusion machinery. Most importantly, the aforementioned tetraspanins (enriched in exosomes) [2] and the small RAB GTPases (e.g., RAB27A, RAB27B, and RAB11) [61,62] are thought to be involved in both the biogenesis and secretion of exosomes. RAB27A RNA interference in melanoma cells was shown to decrease exosome production [63], whereas in HeLa cells, MVB size was strongly increased upon RAB27A RNA interference and redistribution to the perinuclear region occurred upon RAB27B silencing [61]. RAB27A is thought to promote docking of MVBs and fusion to the PM, whereas RAB27B plays a role in vesicle transfer from the Golgi to MVBs and in the mobilization of MVBs to the actin-rich cortex under the plasma membrane. However, RAB27A is not expressed or at very low levels in many cell types, unlike melanoma cells, alluding to the existence of alternate machinery for MVB docking and fusion with the PM. Similarly, RAB11 is also assumed to promote fusion of MVBs to the PM, but rather in response to an increase in cytosolic calcium, as observed in K562 erythroleukaemic cells [61,63].

Finally, a prime physiological role of MVBs is to serve as intermediate vehicles in the degradative lysosomal pathway (Figure 2), in which they fuse with lysosomes. The ILVs within the MVB are then discharged into the lysosomal lumen resulting in degradation of the ILVs and the cargo they potentially carry; this is a particularly important process for limiting activated growth factor signaling [64,65]. Various surface proteins play a key role in the fusion of MVBs with lysosomes, including HD-PTP, the HOP complex, and the GTPase RAB7. Furthermore, the formation of a membrane-fusion system, *i.e.*, soluble NSF attachment protein receptor (SNARE), is required and includes VAMP7, VTL1B, syntaxin 7 and 8 [56,66,67,68].

### 2.2. Ectosome Biogenesis

Ectosomes or SMVs are formed through outward budding of the PM and involves distinctly different mechanisms compared to exosome biogenesis [69]. Generally these vesicles are larger than exosomes, with some overlap in their size distributions, and ectosomes have compositions that lack many of the endosomal features found in exosomes. The formation of ectosomes at the PM primarily involves membrane constituents and their rearrangement, the cytoskeleton, and recruited proteins involved in membrane abscission.

**Figure 4:**
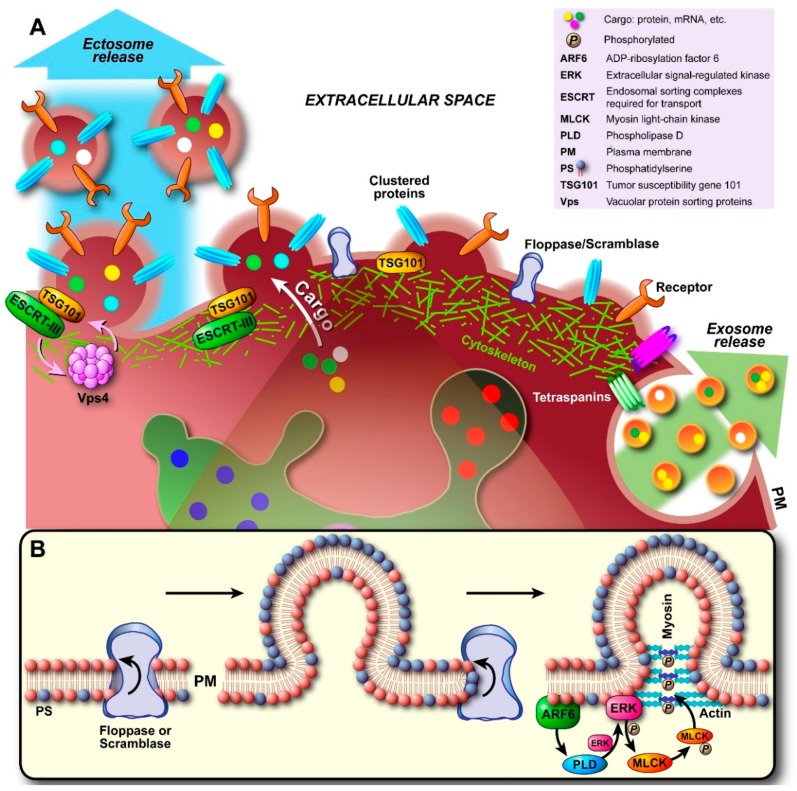
Biogenesis and secretion of ectosomes. (**A**) Initial nucleation at the plasma membrane (PM) starts with clustering of transmembrane proteins and lipids in distinct domains. Recruited and PM associated proteins such as tetraspanins (largely still unidentified) may be involved in sorting of components analogous to exosomal sorting. Additionally, Ca^2+^ release/accumulation and activation of enzymes induce degradation of cytoskeletal components. Outward budding is promoted by externalization of phosphatidylserine (PS) by specific translocases (floppase, scramblase; see also (**B**)). As the cytoskeleton disintegrates locally and becomes more traversable, cytosolic proteins and genetic material are sorted into the lumen. Budding and pinching off are generally thought to occur either through the model proposed in (**B**), where budding involves initiation of a signaling cascade by ARF6 through activation and recruitment of PLD/ERK and phosphorylation of MLCK. This triggers actomyosin contraction and pinching off of the ectosome. Alternatively, recent evidence suggests that recruited TSG101 induces translocation of ESCRT-III to the PM, which in turn results in conical spiral assembly (budding initiator), and finally Vps4 ATPase constriction of the ring of ESCRT-III spirals at the budding neck leads to membrane scission and pinching off, as shown in (**A**).

Upon nucleation (Figure 4A), the interaction between cytoskeletal proteins and the PM is gradually lost, both by a local increase in cytosolic Ca^2+^ and protein degrading enzymes that induce disassembly of the cytoskeleton (e.g., calcium-activated calpains). In this way, an initial delamination of the PM from the cortical cytoskeleton occurs. Concomitantly, lipid translocases, enzymes that are involved in the exchange of lipids between the inner and outer leaflet of the membrane bilayer to maintain membrane asymmetry, are activated to induce changes within the bilayer favoring budding and membrane abscission. In particular, externalization of the phospholipid phosphatidylserine (PS) occurs, which normally exclusively resides in the inner monoleaflet and is actively flipped back to that leaflet by flippases to prevent externalization (PS externalization induces blood clotting and phagocytosis, amongst other events). Although quiescent scramblases [70]—bidirectional lipid translocases that reduce lipid asymmetry—have been alleged to be activated by elevation of cytosolic Ca^2+^, which is clearly associated with cytoskeleton disruption and plasma membrane budding, their exact identity and role in PS externalization during membrane budding remained elusive. Equally, an ATP-driven unidirectional translocase belonging to the floppases was purported to be responsible for the extrafacial enrichment of PS, which drives PM curvature induction and thus vesicle formation (Figure 4B). Concomitantly, flippase activity is attenuated by the influx of free Ca^2+^ ions [71]. Floppases are members of the ATP-binding cassette (ABC) transporter superfamily and especially ABCA1 has been shown to translocate PS; even though cholesterol seems to be its main substrate [72]. However, in human erythrocytes it was shown that vesicle shedding was attenuated when cells were treated with R5421 [73], a scramblase-specific inhibitor [74]. Recent work by Nagata finally identified transmembrane protein 16F (TMEM16F) as the elusive calcium-dependent phospholipid scramblase [75].

Irrespective of whether scramblases, floppases, or both are involved, the unidirectional translocation of PS to the outer leaflet generates a structural imbalance within the lipid bilayer. Consequently, the bilayer bulges in the direction of the outer leaflet (Figure 4B). If translocase activity is high enough, the resulting curvature itself might be sufficient for vesicular fission to occur; analogous to what is known for flippases in the other direction [76]. However, since enzymatic destabilization of cytoskeleton-PM interactions seems to be a major process, proteins that promote cytoskeleton contraction have been implicated to aid in vesicle budding and abscission. In particular the GTP-binding protein ADP-ribosylation factor 6 (ARF6) has been alleged to play a role in ectosome secretion [26]. As shown in Figure 4B, ARF6 initiates a signaling cascade by activating phospholipase D (PLD). Hydrolysis of phosphatidylcholine (PC) by ARF-activated PLD produces membrane-bound phosphatidic acid (PA), which in turn recruits extracellular-signal-regulated kinase (ERK) and molecules that affect vesicle curvature. ERK then phosphorylates myosin light-chain kinase (MLCK), which in turn phosphorylates the myosin light chain and leads to actomyosin contraction and subsequent pinching off of the ectosome.

To complicate matters further, changes in the PM organization may occur via translocase-independent mechanisms, as determined in B lymphocytes [77], and indeed annexin V-negative ectosomes derived from platelets and endothelial cells have also been detected [78,79]. These results suggest that ectosome biogenesis might in some cases proceed whilst lipid asymmetry is maintained and be a direct result of directed cytoskeleton cleavage or the involvement of an abscission machinery. Whilst ESCRT complexes are distinctly associated with processes that occur in endosomes and exosome biogenesis, components of the ESCRT machinery have important functions in PM-associated processes, such as cytokinesis and virus budding. In fact, recent research has shown that ESCRT components may play a key role in the biogenesis of ectosomes at the PM. Nabhan and co-workers showed that budding at the PM is driven by the interaction of the TSG101 subunit of ESCRT-I with the tetrapeptide PSAP motif of arrestin domain-containing protein 1 (ARRDC1); the N-terminal arrestin domain of ARRDC1 directs PM targeting [55]. TSG101 is recruited from its endosomal origin to the PM by the PSAP motif, which is also found in Hrs, and in conjunction with Alix may be involved in the later stages of vesicle budding and fission. The interaction between arrestin-related proteins, TSG101 and Alix in viral budding has been described previously [80]. Finally, ESCRT-III and Vps4 ATPase are recruited and assembled to allow pinching-off of the ectosome and recycling of the vesicle-forming machinery.

To conclude, it is worth mentioning that in particular in aberrant cells, activation of purinergic ATP receptors, protein kinase C, acid sphingomyelinase, p38^MAPK^, and the increase in Ca^2+^, act as initiating and sustaining shedding mechanisms and signals [81,82,83,84,85]. Furthermore, several important problems with regard to ectosome biogenesis are conspicuous and remain to be resolved. First of all, the specific steps and components involved in ectosome biogenesis might be cell-type or function specific and hence various distinct mechanisms have been reported in the literature. The mechanisms by which the cell controls ectosome biogenesis and fate are still elusive. Foremost, even though PS externalization may be required for vesicle budding, this fact might potentially be problematic, since PS externalization has long been known to be an “eat me” signal that initiates phagocytosis, binds C1q and activates complement, and also initiates the blood clotting cascade. In ectosomes from particular cell types, such as platelets (these have the highest scrambling rate known [70]), local shedding of procoagulant ectosomes represents a physiological function that allows assembly of the prothrombinase and tenase complexes, leading to rapid thrombin generation [86] (50- to 100-fold higher procoagulant activity compared with the platelet surface [87]) and therefore is a wanted process. Increased levels of circulating ectosomes from activated platelets on the other hand have been implicated in thrombotic and systemic inflammatory disorders [86,88]. In apoptotic cells and ABs, phagocytosis is a necessary outcome to prevent immunogenic responses and tissue destruction. The question is how initiation of such effects is prevented in ectosomes that have long life-times and distinct functions, such as intercellular signaling. The answer to this question might be multifaceted. First, for phagocytosis to occur, competent cells must be present or recruited via “find me” signals, which might be absent in particular ectosome versions. Second, adapter proteins, recruited during vesicle budding, might in turn recruit proteins that prevent interactions with PS via surface coating. Third, both the number of surface exposed PS molecules and the way that these are presented to recognition receptors on phagocytes determines whether phagocytosis occurs; the same goes for factors involved in blood clotting. However, the critical PS fraction and the way that PS is presented are still largely unknown; multiple PS receptors exist on the surface of phagocytes that bind PS directly, e.g., TIM receptor family (T-cell-transmembrane/immunoglobulin/mucin) and stabilin-2 [89,90], or via bridging molecules such as soluble thrombospondins, milk fat globule epidermal growth factor 8 (MFG-E8), which links to integrins α_v_β_3_ and α_v_β_5,_ or growth arrest-specific gene 6 (Gas6) and protein S (a vitamin K-dependent glycoprotein), which both link to TAM receptors (Tyro-3–Axl–Mer) [91,92,93,94,95]. Additionally, necrotic cells potentially have a significantly higher extrafacial PS fraction compared with apoptotic cells [96], but it has long been known that resting dendritic cells only respond to necrotic and not to apoptotic cells [97]. This suggests that co-factors might be responsible for the initiation of phagocytosis. Fourth, the presence of “don’t eat me” signals, such as CD47, might prevent phagocytosis. In fact, CD47 has recently been detected in exosomes of various origins and ectosomes from human mesenchymal stem cells [98], human platelets [99], and Jurkat cells [100]. Fifth, as described above, annexin V-negative ectosomes have been detected in platelets and endothelial cells [78,79]. Finally, phagocytosis might in particular cases be a wanted outcome for ectosome-based signal transfer. Overall, the exact (and potentially diverse) mechanisms by which a response is elicited toward ectosomes and their processing during their life-time remain to be resolved.

### 2.3. Apoptotic Body Formation

Apoptotic cells undergo a series of distinct changes, such as chromatin condensation, internucleosomal DNA fragmentation, nuclear rupture, mitochondrial swelling and cytochrome c release, proteolytic cleavage of the cytoskeleton and focal adhesion complexes, PS externalization, PM blebbing, disruption of key survival functions, cell shrinkage and commitment to the apoptotic phenotype that all culminate in the packing of the dying cell into ABs, which are then released for phagocytic clearance [41,101,102]. This dismantling of the cell and the formation of ABs is a controlled mechanism to prevent leakage of potentially toxic, enzymatically active or immunogenic components of dying cells into tissues, thereby preventing tissue destruction, inflammation, and autoimmune reactions. The process of AB formation might, however, only be important for large cells that are difficult to engulf in their intact state, since it is known that cells such as neutrophils do not readily form ABs, but are rather phagocytized whole [103,104].

To allow the dismantling of the cell, key structures such as the cytoskeleton need to be weakened. This is predominantly performed by caspases; cysteine proteases designed for protein cleavage rather than degradation, which are normally present within the cell as catalytically inactive zymogens [105]. Effector caspases (caspases-3, -6 and -7) are typically activated through proteolytic cleavage by initiator caspases (caspases-8, -9 and -10) and upon activation initiate cell dismantling. Furthermore, the early stage detachment from the extracellular matrix and cell rounding involves the caspase-dependent dismantling of cell–matrix focal adhesions and cell–cell adhesion complexes. These events are followed by a plethora of events geared toward the demolition stage and AB formation.

In multiple cell types, the outward protrusion that leads to PM blebbing (zeiosis) and ultimately formation of ABs (Figure 1)—blebs are considered to be progenitors of ABs [106]—seems to be driven by local membrane rearrangements that are initiated by caspase‒3-mediated activation of Rho-associated coiled-coil-forming kinase I (ROCK1) [107,108,109] and actin polymerization in the cortical microfilament network. As stated previously, PS externalization, one of the hall marks of apoptosis, serves as an “eat me” signal involving multiple phagocyte surface receptors and results in engulfment and digestion of cellular remains by phagocytic cells [41,102,110,111]. Analogous to ectosome budding, externalization of PS induces a structural imbalance within the lipid bilayer, but in this case, with the size of the bleb, membrane tension alone is insufficient to cause delamination. Unlike ectosome budding, PS externalization during apoptosis is mainly driven by a Ca^2+^-independent scramblase in most cell types. This scramblase was recently determined by Nagata’s group to be the evolutionarily conserved Xk-related protein 8 (Xkr8), which is activated by caspase cleavage (caspase-3 or -7) [112]. In addition, results from the same group implicate the caspase-dependent inactivation of the flippase adenosine triphosphate type 11C (ATP11C) and cell division cycle protein 50A (CDC50A), which is required for PM localization of ATP11C, in apoptotic PS externalization [113].

Bleb formation follows a series of distinct steps, *i.e.*, enucleation, expansion, and retraction, during which cellular debris is packed into the blebs’ lumen and finally pinches off as ABs. Actin polymerization results in the formation of restriction rings where bleb enucleation and formation occur [114,115]. This is achieved through caspase-3-mediated activation of gelsolin, which cleaves actin filaments in a calcium-independent manner [116]. After bleb enucleation, bleb expansion occurs through ROCK1-induced phosphorylation of the myosin light chain, which in turn promotes actomyosin contraction with consequential delamination of the PM from the cortical cytoskeleton membrane. The subsequent blebbing of the PM is a purely physical process that is a result of both the loss of interaction with the cytoskeleton and the increase in hydrostatic pressure due to apoptotic volume decrease (cell shrinkage causes the cytosol to push against the PM and the size of the bleb is proportional to the cortical tension [115]). Although the externalization of PS is scramblase-dependent and ROCK-independent, its subcellular localization during apoptosis is distinctly ROCK-dependent [107,117]. Specifically, the apoptotic blebs become highly enriched with externalized PS and consequently serve as focal recognition points for macrophages to trigger engulfment (*vide supra*). Bleb retraction has been shown to occur ~30 s after bleb initiation and takes nearly 90 s for full completion [118,119]. Retraction is driven by reassembly of the contractile cortex under the bleb membrane. Repetitive cycles of bleb expansion and retraction have been suggested to play a major role in the actual packing of cellular debris into the lumen of the blebs before they pinch off as ABs. However, other mechanisms seem to be involved as well. For instance, actin-myosin clearly plays a central role in apoptotic cellular remodeling, whereas all other cytoskeletal components are dismantled. Recent research, however, shows that rapid dynamic *de novo* assembly of microtubules throughout the cytoplasm aids in packing condensed chromatin into PM blebs, promotes cellular fragmentation, and assist in binding apoptotic cells to phagocytes through extension of rigid spikes [120]. In addition, in late blebs, the condensed chromatin is often surrounded by a cortical layer of endoplasmic reticulum, which might be the result of active translocation and remodeling [106]. Finally, the recent discovery of novel PM protrusions that give rise to ABs shows that AB formation might not be as stochastic as previously assumed; at least in some cell types. For instance, in T lymphocytes, “string-like” membrane protrusions (apoptopodia) are formed after the onset of membrane blebbing and facilitate the separation of blebs into ABs [121]. This formation of apoptopodia (and ABs) in T lymphocytes is negatively regulated by the caspase-activated pannexin 1 (PANX1) channel [122]. In monocytes, a recently discovered “beads-on-a-string” and fragmentation mechanism of AB formation might be involved in facilitating sorting and localization of particular intracellular contents into ABs [123]. These results point in the direction that packing of cellular debris might be a controlled mechanism rather than a random effect due to bleb oscillation. However, more studies are needed to understand this highly regulated process as the formation of ABs is cell type dependent. For instance, the newly discovered “bead-on-a-string” mode of AB biogenesis is not conserved in some of the adherent epithelial cells.

## 3. Extracellular Vesicle Composition

### 3.1. Molecular Composition of Exosomes

Exosomes typically comprise of luminal cargo, *i.e.*, proteins, DNA, RNA, peptides, lipid-derivatives, surrounded by a lipid bilayer membrane (Figure 3B), which serves as a transport vehicle and protects the luminal cargo from the harsh extracellular environment. The luminal contents of exosomes predominantly contain cytosolic proteins derived from the donor cell [20,124]. Interestingly, the composition of the lipid bilayer in exosomes differs from the lipid composition of the PM of the cell of origin [3,125,126]. Development of ExoCarta (Available online: http://www.exocarta.org), a manually curated database that lists proteins, RNA and lipids identified in exosomes [127,128,129], and Vesiclepedia (Available online: http://microvesicles.org), a community annotation compendium for all EVs [130], have allowed researchers to successively deposit identified constituents of exosomes and provide a general overview of their molecular composition [127]. These two databases are regularly supplemented with contributions from different authors working in the EV field. Furthermore, ExoCarta now provides annotations with International Society of Extracellular Vesicles standards thereby aiding researchers in quickly comprehending the characterization done on the exosomes [126].

Since exosomes originate from endosomes, proteins involved in MVB formation (e.g., Alix and TSG101), membrane transport and fusion (e.g., annexins, flotillins, GTPases), adhesion (e.g., integrins), tetraspanins (e.g., CD9, CD63, CD81, CD82), antigen presentation (MHC class molecules), heat shock proteins (HSP70, HSP90) and lipid-related proteins [124,131,132,133] are often identified in exosomes irrespective of the cell type of origin (Figure 3B). Apart from proteins, exosomes are also enriched in particular lipids; primarily ceramide, cholesterol, PS, and sphingolipids [59,134,135]. Interestingly, exosome membranes do not contain lysobisphosphatidic acid (LBPA) [135,136,137], even though LBA has clearly been detected in ILVs and purported to be essential, together with Alix, for their formation [138]. As pointed out by Brouwers *et al.*, LBPA might play an exclusive role in the formation of lysosomally targeted MVBs rather than in exosome-generating MVBs [136]. Additionally, the discrepancies in lipid composition between these types of MVBs may also suggest that their formation is strictly controlled and a significantly higher degree of cellular control over EVs and their fate might be present as currently thought. Exosomes also contain detergent-resistant domains in their lipid membrane, *i.e.*, lipid rafts. These rafts are not only enriched in the aforementioned lipids, but also various proteins such as flotillins seem to accumulate in lipid rafts and not surprisingly, lipid rafts have been implicated in exosome biogenesis. 

Exosomes also have polysaccharide and glycan signatures on their outer surface, predominantly comprising of mannose, α-2,3- and α-2,6-sialic acids, complex *N*-linked glycans, and polylactosamine [139,140]. Exosomes have been reported to carry RNA, including mRNAs, miRNAs and some non-coding RNAs [11]. Considering the fact that ILVs bud into the MVBs by invagination of the limiting membrane of MVBs in the cytosol, this invagination also sequesters a considerable amount of cytosol, including the therein contained proteins and RNA. Whilst exosomes contain a common set of proteins irrespective of the cell type (some of which are presumably involved in exosome biogenesis), recent studies have shown a tissue/cell type-specific signature in exosomes [133]. It is unclear how these proteins are targeted to exosomes. More studies are needed to unravel any sorting/packaging signals in exosomes and address the question of selectivity *versus* randomness.

### 3.2. Molecular Composition of Ectosomes

**Figure 5:**
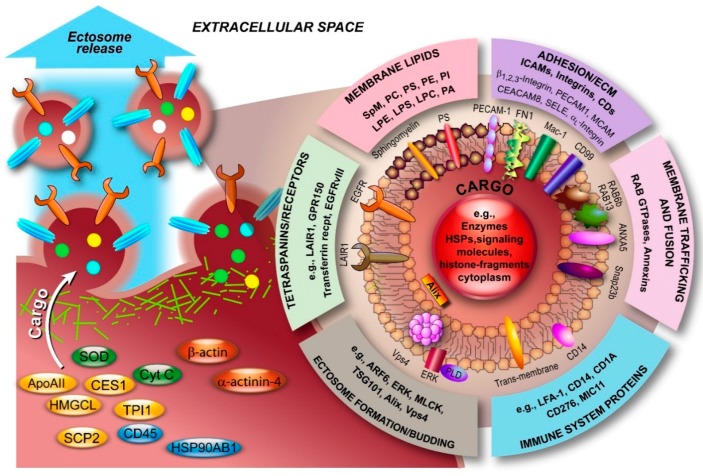
Molecular composition of ectosomes. Ectosomal membranes consist of various classes of lipids. Furthermore, in recent years, numerous components with diverse functions have been identified, predominantly from blood, immune and endothelial cells, and atherosclerotic plaques. The overview serves to illustrate this diversity and is far from complete. Data was retrieved from Vesiclepedia (Available online: http://www.microvesicles.org). ANXA5, annexin A5; ApoAII, apolipoprotein AII; ARF6, ADP-ribosylation factor 6; CD45, protein tyrosine phosphatase; CEACAM8, carcinoembryonic antigen-related cell adhesion molecule 8; CES1, carboxylesterase 1; Cyt C, cytochrome C; ECM, extracellular matrix; EGFRvIII, mutated form of epidermal growth factor receptor; ERK, extracellular-signal-regulated kinase; FN1, fibronectin 1; GPR150, G protein-coupled receptor 150; HMGCL, 3-hydroxymethyl-3-methylglutaryl-coenzyme A lyase; HSP90AB1, heat shock protein 90 kDa alpha (cytosolic) class B member 1; LAIR1, leukocyte-associated immunoglobulin-like receptor 1; LFA-1, lymphocyte function-associated antigen 1; LPC, lysohosphatidylcholine; LPE, lysophosphatidylethanolamine; LPS, lysophosphatidylserine; Mac-1, macrophage-1 antigen; MCAM, melanoma cell adhesion molecule; MLCK, myosin light-chain kinase; PA, phosphatidic acid; PC, phosphatidylcholine; PE, phosphatidylethanolamine; PECAM1, platelet/endothelial cell adhesion molecule 1; PI, phosphatidylinositol; PLD, phospholipase D; PS, phosphatidylserine; SCP2, sterol carrier protein 2; SELE, selectin E; Snap23b, synaptosomal-associated protein 23b; SOD, superoxide dismutase; SpM, sphingomyelin; TPI1, triosephosphate isomerase 1.

Ectosomes are relatively heterogeneous, both in size and in their composition. Like exosomes, ectosome membranes are not identical to the PM of the cell of origin, but rather specific changes are induced upon nucleation and budding of the PM that cause this discrepancy. Although ectosomes contain similar types of cargo as exosomes, the molecular composition of ectosomes is less well defined compared with exosomes. Nevertheless, an increasing number of studies have led to a significant number of entries into the aforementioned databases. Several studies have highlighted the fact that ectosomes contain a diverse population of proteins (see also Figure 5), including matrix metalloproteinases (MMPs) [28,29,30,31], glycoproteins, e.g., GPIb, GPIIb-IIIa and P-selectin [32,33,34,35], integrins, e.g., Mac-1 [35,36], receptors, e.g., EGFRvIII [141], and cytoskeletal components such as β-actin and α-actinin-4 [142]. In fact, proteomic analysis of monocytic THP-1 cell-derived ectosomes, predominantly in the range of 780‒990 nm, by Bernimoulin and co-workers revealed distinct expression patterns involving 1076 proteins upon different stimuli and 100 proteins that were commonly present, including cytoskeletal components, adhesion receptors, signaling molecules, and mitochondrial proteins [142]. Similarly, Keerthikumar *et al.* confirmed the enrichment of mitochondrial, centrosomal and ribosomal proteins in ectosomes by proteomic analysis [22]. The analysis also confirmed the depletion of ESCRT proteins, tetraspanins and proteins implicated in fusion and trafficking (e.g., annexins, integrins and flotillins). Interestingly, the study also highlighted an exclusive set of RAB GTPases that is enriched in exosomes and ectosomes, at least in neuroblastoma cells. Minciacchi *et al.* also reported a distinct cargo in large oncosomes and showed that particularly mitochondrial proteins were enriched [25]. Recently, Lunavat and collaborators described the existence of unique RNA cargo in ectosomes [143]. Weerheim *et al.* determined that circulating ectosome membranes, next to PS (3.63%), which is involved in vesicle budding, predominantly contained phosphatidylcholine (PC; 59.2%), sphingomyelin (20.6%), and also phosphatidylethanolamine (PE; 9.4%) [144]. However, lysophospholipids were also detected (<2%/class). Additionally, an extensive lipidomic analysis, including fatty acyl moiety evaluation, was recently performed by Losito and co-workers [145]. The examples presented in Figure 5, which serves to illustrate that ectosomes are as diverse in composition as exosomes, represent only a fraction of the identified components. What complicates the unperturbed assignment of components to ectosomes is the fact that a diverse nomenclature is used in the literature for various vesicles, both based on their origin and size. Whilst exosomal vesicles have been relatively easy to identify based on their size and the term “exosome” is widely used, vesicles larger than exosomes have been classified almost ambiguously and this affects both ectosomes and ABs. Consequently, assignment of components to ectosomes should be taken with care.

### 3.3. Molecular Composition of Apoptotic Bodies

Although apoptosis has been the subject of intense research over the past decades, apoptotic remnants of cells have long been regarded as “garbage bags”. That relatively little is known about the molecular composition of ABs might be a result of their size heterogeneity, diverse cell origins, the diverse apoptosis triggers involved, and the premise that ABs are randomly stuffed with cellular debris. A quick look in the literature and Vesiclepedia provides little evidence for structural molecular composition studies and indicates that a thorough characterization is urgently needed. Naturally some information is already available, but it is rather locked in various publications on apoptosis and deposition of the information by the authors into compendiums such as Vesiclepedia might provide an initial basis for further characterization studies.

Nonetheless, a study by Mallat *et al.* showed that ABs of human monocytic and lymphocytic origin from atherosclerotic plaques were enriched with PS, coagulation factor III, and annexin A5 (a phospholipase A_2_ and protein kinase C inhibitory protein with calcium channel activity) [146]. A more recent proteomic evaluation of thymocyte-derived ABs in BALB/c mice identified 142 different proteins, including a myriad of heat shock, histone-related and cytosolic proteins, (pseudo)oncogenes, and proteins with immunological relevance [147]. Shotgun proteomics of ABs from human biliary epithelial cells identified 11 distinct proteins, including annexin A6, heat shock protein β6, low-density lipoprotein receptor-related protein 1, and RAB11A [148]. The identified proteins were largely involved in (auto)immune reactions such as nuclear factor kappa B (NF-κB) activation, ERK and Notch signaling pathways, and IL8- and CXCR2-mediated signaling events. More recently, a total of 1028 proteins were differentially abundant between whole apoptotic sample and apoptotic body-enriched samples [123]. The study highlighted the marked depletion of nuclear components in ABs.

## 4. Extracellular Vesicle Isolation Methods

Isolation of the various classes of EVs is generally performed by strategic purification of rough isolates from cell cultures, cell suspensions, tissues, and body fluids. Isolation methods exploit the physical properties of EVs, in particular their buoyant densities, size, and surface composition, and include ultra-centrifugation, density gradient centrifugation, gel filtration, polymer-based precipitation, immuno-affinity methods, filtration, and flow field–flow fractionation [149,150,151,152,153]. However, with the available techniques, it is currently impossible to separate any single EV subtype devoid of other EV subpopulations to homogeneity [22,24]. Consequently, reports on experimental results from allegedly purified EVs need to be considered with caution.

### 4.1. Exosome Isolation Methods

The most commonly used method for isolating exosomes is ultracentrifugation at 100,000–120,000× *g* [8,21,154]. However, the major disadvantage of using a series of differential centrifugation steps coupled with ultracentrifugation is its inefficiency in separating EV subtypes [40]. To avoid co-isolation of EV subtypes, researchers utilize filtration (0.1 or 0.2 μm pore size) and/or perform differential centrifugation (medium speed 10,000× *g*) that sediment larger EVs including ectosomes and ABs [26]. Ultrafiltration and microfiltration have been utilized to rapidly isolate exosomes from urine [155,156]. Multiple studies have highlighted the fact that ultracentrifugation can be used in conjunction with other isolation methods including density gradient centrifugation (sucrose, sucrose-deuterium oxide (D_2_O), and OptiPrep™ (iodixanol) [154], which separates exosomes according to their buoyant density. Based on the purity of the exosome preparation, density gradient separation is the best enrichment technique that is currently in use [21]. Whilst optimal exosome isolation can be achieved through density gradient centrifugation, the technique requires more sample, is tedious and time consuming. In addition to density gradient centrifugation, immunoaffinity based methods (immunobeads and FACS), have also been utilized to isolate exosomes. Multiple exosomal membrane molecules have been used for this purpose including EPCAM, CD63, CD9, HER2 and A33. While this method can be robust, one of the inherent problems with immunocapture techniques is that the negative population (EPCAM negative when EPCAM immune beads are used) is often ignored [40]. In addition, non-specific protein binding can also confound the interpretation of the results [21,154].

With the increasing interest in the physiological and pathological roles of exosomes, many commercial kits that allow “easy and quick isolation procedures” are now routinely developed and are available for use. While most of these kits isolate/precipitate exosomes, the kits invariably suffer from co-isolation of other EVs and protein complexes. Hence, we emphasize caution while interpreting data obtained from precipitation kits [157]. On the other hand, the commercial kits are robust, fast, use very little sample and therefore serve as ideal choice for identification of exosome-related disease biomarkers. Currently, there is no gold standard method for isolating exosomes and, hence, the method of choice should be determined based on the (patho)biological question of interest.

### 4.2. Ectosome Isolation Methods

Since the physical differences between exosomes and ectosomes are relatively small and a significant overlap occurs as far as their sizes are concerned, separation of these two classes of EVs is relatively difficult. For the isolation of ectosomes, the same strategies may be employed as for exosomes (*vide supra*). However, since ectosomes have distinctly different surface compositions, isolation based on these specifics through affinity-based methods might be the best strategy to obtain enriched fractions. Unfortunately, membrane antigens that can reliably serve as markers of ectosomes are currently non-existent.

### 4.3. Studying Apoptotic Bodies

Generally, apoptotic bodies are not isolated as other EVs are, but are rather studied in well-defined cell models of apoptosis. Nonetheless, when it is necessary to isolate ABs from cell cultures or body fluids, differential centrifugation may be employed. Since ABs are large, they easily sediment at low g values. A general approach would start with a low speed spin at ~300−500× *g* to remove cells, followed by a short centrifugation of the rough isolate at ~1000× *g* to remove cellular debris, followed by a longer centrifugation at higher g forces (~10,000 < x < ~16,000× *g*) to obtain the AB fraction [152,158,159]. Further AB purification steps such as immunoaffinity purification or filtration might be necessary depending on the goal of the isolation and the question under investigation. Moreover, unlike exosomes and ectosomes, ABs may not be stable for longer time periods and hence harsh isolation methods cannot be utilized.

### 4.4. General Isolation Problems

Besides the aforementioned specific problems, isolation procedures suffer from a number of general restrictions. First of all, since centrifugation protocols are not standardized, discrepancies within differential centrifugation protocols invariably lead to inconsistencies in the isolates. This fact may, at least partially, explain the differences in biological effects of EVs reported by various research groups. Second, an inherent problem with isolating exosomes and ectosomes from body fluids is the fact that these potentially contain high amounts of non-EV particles such as lipoproteins, viruses, and aggregate-forming (bio)molecules. Exosomes generally overlap in size with viruses and lipoproteins, whereas ectosomes overlap with the size range of bacteria. Such contaminants need to be removed in order to obtain sufficiently pure isolates. With regard to viruses, sucrose gradients are inefficient at separating them from exosomes, but Cantin *et al.* recently showed that the use of iodixanol gradients do allow their separation and purification [160]. Contamination with proteins and protein complexes, such as insoluble immune complexes, also perturb the isolation of both exosomes and ectosomes [161,162]. Furthermore, filtration of EV isolates under pressure to remove particular contaminants or fractions carries the inherent risk that fragmentation of vesicles occurs and thus sample might be lost. Finally, specific purification protocols after ultracentrifugation not only affect EV purity and yield, but often lead to protein loss in the preparation [163,164]. However this loss of protein does not correlate with loss of vesicles. With regard to vaccination, the method of purification is therefore extremely important to take note of when comparing manuscripts from different research groups.

Isolation of EVs for diagnostic purposes in a clinical setting currently has a number of distinct limitations. These include the time required for isolation and analysis and a potential lack of suitable infrastructure. Recently, Sáenz-Cuesta and co-workers compared various protocols with regard to urine and blood EV samples [153]. They conclude that any method used should be compatible with the simple infrastructure found in general clinical laboratories, where apparatus such as ultracentrifuges are not readily available, allow isolation of EVs with high accuracy and in sufficiently high concentrations, permit isolation of both small and large EVs, and validation of the isolation procedure should be performed by a group with significant expertise in the EV research field. Furthermore, the workflow from sample collection to EV characterization would require standardization to allow a direct comparison between clinical diagnostic labs. Overall, a medium-speed differential centrifugation protocol would currently be most suitable in a clinical setting [153].

For further information on currently available isolation methods, their strengths and caveats, and their impact on the quality of the final isolates, the reader is referred to several excellent and comprehensive reviews [149,152,153].

## 5. Function of EVs and Development of EV-Based Technologies

Although the exact physiological functions of EVs are poorly understood, when generalizing for all classes of EVs, these all function as transport vehicles of some sort. Exosomes have been shown to contain molecules, predominantly from an endosomal and cytosolic origin, for intercellular communication over a short range. Ectosomes contain ubiquitous cargo and are believed to also be involved in cell-cell communication, whereas ABs function to transport and present cellular debris from intentional cell suicide to phagocytic cells for further dismantling and recycling of building-blocks. Furthermore, increasingly evidence accumulates that cells modify the content of EVs in response to extrinsic stressors such as heat shock, hypothermia, hypoxia, oxidative stress, and infectious agents. These results suggest that the EVs are connected to intracellular signaling and are part of the global intricate mechanism to maintain physiological homeostasis; the levels of which we are just beginning to understand. It also suggests that perturbation of the roles that EVs play in homeostasis potentially results in disease and a link can indeed be established between EVs and various diseases. Consequently, EVs have also become of interest with regard to their pathophysiology, the development of novel therapeutic modalities, and because particularly exosomes are ubiquitously present in bodily fluids, exosomes are deemed ideal as diagnostic biomarkers. In this focus edition, Iraci and co-workers provide an extensive overview of the physiological roles of EVs and their signaling properties [44].

Tumor cells have been reported to secrete increased amounts of exosomes [165]. Since these tumor-derived exosomes carry the tumor-specific genomic and proteomic signatures, tumor-derived exosomes are ideal and unique targets for cancer detection. However, the fact that tumor-derived exosomes carry the hallmark properties for tumorigenicity also means that these exosomes might aggravate the tumorigenic potential already present in cells [133]. Indeed a number of studies seem to confirm that exosomes secreted by tumor cells play a role in the growth and dissemination of tumor cells [166,167,168,169,170]. For instance, Lázaro-Ibáñez *et al.* recently showed that the various prostate cancer cell-derived EVs subgroups carried different fractions of genomic DNA (gDNA) fragments of *MLH1*, *PTEN*, and *TP53* genes, including mutations [158]. Their results suggest that nucleic acids are selectively and cell-dependently packed into the various EV subtypes and that circulating EVs potentially contribute to both pre-metastatic niche formation and tumor metastasis. On the other hand, some investigations report quite the opposite, *i.e.*, anti-tumorigenic properties, such as tumor cell apoptosis induction in pancreatic carcinoma or enhancement of anti-tumor immunity [171,172]. Furthermore, even if tumor-derived EVs are found in the circulation of cancer patients, this must not necessarily mean that EVs are actively involved in tumor progression, but could simply be the result of tumor expansion and thus enhanced EV secretion. Nonetheless, tumor-derived EVs show both the potential as cancer biomarkers as well as the possibility to develop novel anti-cancer therapeutics. In this focus edition, Ciardiello *et al.* discuss the current state of the art regarding the EV-cancer connection [43], whereas Ohno and Kuroda focus on the development of EV-based therapeutics [45].

Similarly, EVs have drawn the attention of researchers investigating degenerative brain disorders, such as Alzheimer’s dementia and Parkinson’s disease, ischemic stroke, neuro-inflammation, and epilepsy. However, the study of EVs of neuronal origin in neurological disorders is still challenging due to technical and ethical limitations; *in vivo* sampling of brain material cannot readily be performed, apart from biopsies for diagnostic purposes, and repetitive sampling of cerebrospinal fluid is overall considered unethical, but still recent research results from brain tumors seem to be promising. For instance, Skog *et al*. showed that nested-PCR-based detection of the tumor-specific epidermal growth factor receptor EGFRvIII transcript in serum-purified exosomes allows diagnosis of a glioblastoma sub-set [170]. Overall, particularly exosomes are implicated to facilitate the spread and accumulation of key disease-causing neuronal proteins, such as β-amyloid [173,174,175] and α-synuclein [176,177,178]. Here, Vella and co-workers review the role of exosomes in protein trafficking with respect to Alzheimer’s and Parkinson’s disease and not only highlight recent advances but also the remaining challenges [46].

EVs have been shown to be secreted by stem cells, which in itself is not surprising given their undifferentiated nature and the potential that stem cells carry. The fact that stems cells are the “mother of all cells” and potentially can produce any cell type, stem cell therapy has been heralded as the ultimate regenerative therapy. However, the results from various experimental and clinical studies have not produced the expected results for multiple reasons. It is known that stem cells secrete a myriad of biomolecules in order to communicate with the cells in the surrounding tissue. Consequently, researchers tried to determine the factors involved, but no single biomolecule or combination could induce the desired therapeutic effects of stem cell transplantation. Since EVs are involved in intercellular communication and may contain all the signals required for successful communication, even at multiple levels and via multiple pathways, EVs have attracted the attention of researchers in the stem cell therapeutics field. Stem-cell derived EVs might themselves constitute potent therapeutics against various degenerative diseases. Recent research already validates this assumption, since various groups have found encouraging results from various stem cell types, e.g., mouse embryonic stem cells EVs enhanced survival and expansion of hematopoietic progenitor cells [179], endothelial progenitor cells-derived EVs protect against angiotensin II-induced cardiac hypertrophy [180], and mesenchymal stem cell EVs reduce infarct size in a mouse model of myocardial ischemia/reperfusion injury [181]. Focusing on the effects of EVs, Zhang and colleagues briefly review the current advances in the stem cell therapeutics field [47].

Some indications that ABs are not just garbage bags advertising “eat me” signals to phagocytic cells, but rather might have more intricate roles, both positive and negative ones, come from recent research. Kogianni and co-workers showed that osteocyte ABs were able to initiate *de novo* osteoclastic bone resorption on quiescent bone surfaces *in vivo*, which suggests a physiological signaling role of ABs in directed osteocyte apoptosis in damaged bone [182]. Phagocytosis of HepG2-derived ABs by hepatic stellate cells (HSC) activates JAK1/STAT3 and, to a lesser extent, PI3K/Akt/NF-κB survival pathways, upregulating Mcl-1 and A1 anti-apoptotic proteins, which leads to HSC survival and propagation of liver fibrosis [183]. That ABs can be used in a therapeutic setting was recently demonstrated by Marin-Gallen *et al.* [184]. These authors showed that tolerogenic dendritic cells (DCs) could be generated that reestablished peripheral tolerance in type 1 diabetes by pulsing DCs *in vitro* with ABs from β cells. Consequently, treated DCs diminished the expression of the co-stimulatory molecules CD40 and CD86 and reduced secretion of proinflammatory cytokines, thereby reducing autoimmunity towards β cells and thus insulitis. Furthermore, Schiller and co-workers observed that active packing of immunogenic molecules into ABs occurred early during apoptosis, well before DNA degradation [185]. These results indeed suggest that formation of ABs might follow a distinct “plan” and thus a significant level of control by the cell might be present.

Finally, the positive and negative modulation of the immune response by both immune and non-immune cell-derived EVs is one of the best established (patho)physiological functions of EVs. Exosomes have been shown capable of direct antigen presentation since they preserve the topology of the antigen-presenting cell (APC) from which they originate and directly stimulate CD8^+^ and CD4^+^ T cells through surface MHC-I and II molecules [186,187]. Exosomes have also been shown to be involved in indirect antigen presentation either through transfer of antigenic peptides to APCs [188,189] or by cross-dressing APCs [188,190,191]. Not surprisingly, EVs have been shown to carry a variety of antigens from various origins, including the aforementioned tumor-derived antigens, pathogens-derived antigens, e.g., antigens from *Cytomegalovirus* [192] or *Mycobacterium bovis* bacillus Calmette-Guérin [193], and B cell-derived antigens [194,195]. Besides the tolerogenic effect elicited by ABs through DC modulation, epithelial cells of the small intestine have been shown to release MHC class II^+^ exosome-like structures, called “tolerosomes”, which induce specific tolerance to orally administered antigen ovalbumin [196]. Lastly, recent research suggests that EVs not only transfer antigens to APCs, but also signals that induce transformation of recipient cells into immunogenically competent APCs [197]. Notwithstanding these important results, it is imperative to emphasize that the majority of results to date have been derived from *in vitro* experiments on immune cells or lab animals treated with *in vitro* purified EVs. A significant gap exists between the knowledge gained from these experiments and the potential *in vivo* immunomodulatory roles of EVs, especially in humans. Nonetheless, as our understanding of the roles that EVs play in immune regulation develops, new therapeutic options will certainly become available that might allow inhibition of tumor-derived EVs and modulation of the tumor microenvironment, modification of the release of endogenous immunosuppressive EVs, or even specifically engineered EVs as novel therapeutics. In this focus edition, Ohno and Kuroda discuss the potential of EV-based therapeutics [45], whereas comprehensive reviews covering the role of EVs in immune system-related processes were recently provided by Robbins and Morelli [198] and Théry *et al.* [124].

## 6. Current Issues in EV Research

Although a significant amount of knowledge regarding EVs has been accumulated over the past few years and researchers in various fields of life sciences have turned their attention to EVs, the field is still nascent and faces a number of potential hurdles:

1. Owing to the characteristics of EVs, past studies named them based on the sample source thereby creating multiple names, e.g., ectosomes have aliases such as exosome-like vesicles, shedding vesicles, microvesicles, nanoparticles, microparticles, and oncosomes. These names, apart from a lack of uniform use, are often misleading. For instance, the term “nanoparticle” is normally reserved for solid particulate matter with a size below 100 nm in at least one dimension, e.g., silver colloidal nanoparticles, carbon nanoneedles, *etc.* Hence, there is an urgent need to perform standardization of EV nomenclature [40]. However, such a standardization is also only possible when genuine and unique markers for different types of EVs can be identified [24]. Furthermore, a potential conflict exists with respect to the term “exosome” since this term is also used to denote a multi-protein complex that contains multiple 3′→5′ exoribonucleases and is involved in the degradation of various types of RNA [199].
2. Robust isolation methods that do not compromise on the purity of the isolate are required in order to exploit EVs in biomedical research and therapeutics.
3. The size distribution of vesicles released by apoptotic cells has not yet been systematically investigated.
4. Linked to the aforementioned problems is the fact that the various size-ranges used by researchers to denote the EV they are investigating is extremely heterogeneous. There is a need for consensus on the size-ranges that typify each form of EV.
5. With the current strategies to purify EV types, it is impossible to assess how various types of EVs interact and produce a synergistic and/or antagonistic effect. Consequently, the exclusion of particular EV types from experiments might lead to loss of relevant information regarding EVs in general (synergistic, antagonistic, interconnected networks?).
6. The exact mechanisms involved in the biogenesis of EVs have not yet been fully elucidated. It is also largely unknown whether packaging of cargo into exosomes, ectosomes, and ABs and their secretion into the extracellular space is a selective or a random process; although some recent data is emerging that suggests some measure of cellular control.
7. Multiple studies have highlighted the functional roles of EVs *in vitro* using variable concentrations of EVs. Despite this acquired knowledge*,* very little is known about the stoichiometry of EVs and the most relevant physiological concentrations of EVs. Equally, little is known about their half-lives in tissues and organs. However, several studies have found that the half-life of EVs in circulation is approximately 1.5–3 min [140,200,201,202].
8. It is still unclear why EVs are abundant (at least based on the detection of enriched proteins such as Alix and TSG101) in bodily fluids that can be secreted (e.g., breast milk, saliva and urine) and relatively depleted in internal bodily fluids (e.g., blood, cerebrospinal fluid).
9. Very little is known about the physiological role of EVs and their contribution to homeostasis, which makes it virtually impossible to understand their pathobiological role and develop safe and effective therapeutic interventions.
10. The question also arises why all types of EVs are released by apoptotic cells, and what their interplay is. Additionally, why are different fractions of gDNA fragments from deranged cells packed into the various EV types?
11. The underlying mechanism of how EVs communicate with the target cells and how selectivity is achieved is poorly understood. Understanding this is again a prerequisite to develop effective therapeutics that target this communication and for the development of engineered exosome-derived therapeutic vehicles.
12. Finally, cells modulate the composition of EVs in response to exogenous stress. Understanding the mechanisms involved might lead to the development of therapeutics that exploit this property.

## 7. Conclusions

Extracellular vesicles are highly specific and multi-purpose vehicles that are purported to be involved in vast intercellular communication and/or biomolecule (mass) transfer networks. Some researchers have compared their function with the extension of the borders of the cell of origin to the distant target cell. One major advantage over secreted signaling molecules is the fact that EVs deliver their signal at great distances without dilution or degradation, since the biomolecules are securely transferred within their capsule. Furthermore, various cargos are not only selectively delivered to the target cell, but also potentially to specific structures within that cell, e.g., the target cell’s PM. However, disruption of their normal function may lead to disease. In this sense the correct size and composition may play key roles in whether the particular EV involved plays a physiological role or a pathological one. It is perhaps this fact that is responsible for the Janus-faced results that have been obtained. One is tempted to assume that the existence of EVs, at least in part, might be one reason why so many diseases have eluded us thus far. Understanding their physiological roles and the factors that induce the switch to a pathological role are important when developing novel therapeutic strategies. However, much still remains to be discovered, since we have just scratched the surface of the enigma called “extracellular vesicle”.

## References

[1] Raposo G., Stoorvogel W. (2013). Extracellular vesicles: Exosomes, microvesicles, and friends. J. Cell Biol..

[2] Simons M., Raposo G. (2009). Exosomes-vesicular carriers for intercellular communication. Curr. Opin. Cell Biol..

[3] Balaj L., Lessard R., Dai L., Cho Y.-J., Pomeroy S.L., Breakefield X.O., Skog J. (2011). Tumour microvesicles contain retrotransposon elements and amplified oncogene sequences. Nat. Commun..

[4] Cossetti C., Iraci N., Mercer T.R., Leonardi T., Alpi E., Drago D., Alfaro-Cervello C., Saini H.K., Davis M.P., Schaeffer J. (2014). Extracellular vesicles from neural stem cells transfer IFN-γ via Ifngr1 to activate Stat1 signaling in target cells. Mol. Cell.

[5] Théry C., Zitvogel L., Amigorena S. (2002). Exosomes: Composition, biogenesis and function. Nat. Rev. Immunol..

[6] Gangoda L., Boukouris S., Liem M., Kalra H., Mathivanan S. (2015). Extracellular vesicles including exosomes are mediators of signal transduction: Are they protective or pathogenic?. Proteomics.

[7] Keller S., Ridinger J., Rupp A.-K., Janssen J., Altevogt P. (2011). Body fluid derived exosomes as a novel template for clinical diagnostics. J. Transl. Med..

[8] Théry C., Amigorena S., Raposo G., Clayton A. (2006). Isolation and characterization of exosomes from cell culture supernatants and biological fluids. Curr. Protoc. Cell Biol..

[9] Lässer C., Alikhani V.S., Ekström K., Eldh M., Paredes P.T., Bossios A., Sjöstrand M., Gabrielsson S., Lötvall J., Valadi H. (2011). Human saliva, plasma and breast milk exosomes contain RNA: Uptake by macrophages. J. Transl. Med..

[10] Pan B.-T., Johnstone R.M. (1983). Fate of the transferrin receptor during maturation of sheep reticulocytes *in vitro*: Selective externalization of the receptor. Cell.

[11] Valadi H., Ekström K., Bossios A., Sjöstrand M., Lee J.J., Lötvall J.O. (2007). Exosome-mediated transfer of mRNAs and microRNAs is a novel mechanism of genetic exchange between cells. Nat. Cell Biol..

[12] Samuel M., Bleackley M., Anderson M., Mathivanan S. (2015). Extracellular vesicles including exosomes in cross kingdom regulation: A viewpoint from plant-fungal interactions. Front. Plant Sci..

[13] Guescini M., Genedani S., Stocchi V., Agnati L.F. (2010). Astrocytes and Glioblastoma cells release exosomes carrying mtDNA. J. Neural Transm..

[14] Kahlert C., Melo S.A., Protopopov A., Tang J., Seth S., Koch M., Zhang J., Weitz J., Chin L., Futreal A. (2014). Identification of double-stranded genomic DNA spanning all chromosomes with mutated KRAS and p53 DNA in the serum exosomes of patients with pancreatic cancer. J. Biol. Chem..

[15] Thakur B.K., Zhang H., Becker A., Matei I., Huang Y., Costa-Silva B., Zheng Y., Hoshino A., Brazier H., Xiang J. (2014). Double-stranded DNA in exosomes: A novel biomarker in cancer detection. Cell Res..

[16] Lee T. H., Chennakrishnaiah S., Audemard E., Montermini L., Meehan B., Rak J. (2014). Oncogenic *ras*-driven cancer cell vesiculation leads to emission of double-stranded DNA capable of interacting with target cells. Biochem. Biophys. Res. Commun..

[17] Rechavi O., Goldstein I., Kloog Y. (2009). Intercellular exchange of proteins: The immune cell habit of sharing. FEBS Lett..

[18] Baluska F., Volkmann D., Barlow P.W. (2004). Cell bodies in a cage. Nature.

[19] Carrington J.C. (2000). RNA silencing. Moving targets. Nature.

[20] Mathivanan S., Ji H., Simpson R.J. (2010). Exosomes: Extracellular organelles important in intercellular communication. J. Proteom..

[21] Kalra H., Adda C.G., Liem M., Ang C.S., Mechler A., Simpson R.J., Hulett M.D., Mathivanan S. (2013). Comparative proteomics evaluation of plasma exosome isolation techniques and assessment of the stability of exosomes in normal human blood plasma. Proteomics.

[22] Keerthikumar S., Gangoda L., Liem M., Fonseka P., Atukorala I., Ozcitti C., Mechler A., Adda C., Ang C.-S., Mathivanan S. (2015). Proteogenomic analysis reveals exosomes are more oncogenic than ectosomes. Oncotarget.

[23] Pan B.-T., Teng K., Wu C., Adam M., Johnstone R.M. (1985). Electron microscopic evidence for externalization of the transferrin receptor in vesicular form in sheep reticulocytes. J. Cell Biol..

[24] Lotvall J., Hill A.F., Hochberg F., Buzas E.I., di Vizio D., Gardiner C., Gho Y.S., Kurochkin I.V., Mathivanan S., Quesenberry P., Sahoo S. (2014). Minimal experimental requirements for definition of extracellular vesicles and their functions: A position statement from the International Society for Extracellular Vesicles. J. Extracell. Vesicles.

[25] Minciacchi V.R., You S., Spinelli C., Morley S., Zandian M., Aspuria P.J., Cavallini L., Ciardiello C., Reis Sobreiro M., Morello M. (2015). Large oncosomes contain distinct protein cargo and represent a separate functional class of tumor-derived extracellular vesicles. Oncotarget.

[26] Muralidharan-Chari V., Clancy J., Plou C., Romao M., Chavrier P., Raposo G., D'Souza-Schorey C. (2009). ARF6-regulated shedding of tumor cell-derived plasma membrane microvesicles. Curr. Biol..

[27] Stein J.M., Luzio J.P. (1991). Ectocytosis caused by sublytic autologous complement attack on human neutrophils. The sorting of endogenous plasma-membrane proteins and lipids into shed vesicles. Biochem. J..

[28] Gasser O., Hess C., Miot S., Deon C., Sanchez J.C., Schifferli J.A. (2003). Characterisation and properties of ectosomes released by human polymorphonuclear neutrophils. Exp. Cell Res..

[29] Mochizuki S., Okada Y. (2007). ADAMs in cancer cell proliferation and progression. Cancer Sci..

[30] Li C.J., Liu Y., Chen Y., Yu D., Williams K.J., Liu M.L. (2013). Novel proteolytic microvesicles released from human macrophages after exposure to tobacco smoke. Am. J. Pathol..

[31] Martinez de Lizarrondo S., Roncal C., Calvayrac O., Rodriguez C., Varo N., Purroy A., Lorente L., Rodriguez J.A., Doeuvre L., Hervas-Stubbs S. (2012). Synergistic effect of thrombin and CD40 ligand on endothelial matrix metalloproteinase-10 expression and microparticle generation *in vitro* and *in vivo*. Arterioscler. Thromb. Vasc. Biol..

[32] Del Conde I., Shrimpton C.N., Thiagarajan P., Lopez J.A. (2005). Tissue-factor-bearing microvesicles arise from lipid rafts and fuse with activated platelets to initiate coagulation. Blood.

[33] Heijnen H.F., Schiel A.E., Fijnheer R., Geuze H.J., Sixma J.J. (1999). Activated platelets release two types of membrane vesicles: Microvesicles by surface shedding and exosomes derived from exocytosis of multivesicular bodies and α-granules. Blood.

[34] Falati S., Liu Q.D., Gross P., Merrill-Skoloff G., Chou J., Vandendries E., Celi A., Croce K., Furie B.C., Furie B. (2003). Accumulation of tissue factor into developing thrombi *in vivo* is dependent upon microparticle P-selectin glycoprotein ligand 1 and platelet P-selectin. J. Exp. Med..

[35] Mezouar S., Darbousset R., Dignat-George F., Panicot-Dubois L., Dubois C. (2015). Inhibition of platelet activation prevents the P-selectin and integrin-dependent accumulation of cancer cell microparticles and reduces tumor growth and metastasis *in vivo*. Int. J. Cancer.

[36] Pluskota E., Woody N.M., Szpak D., Ballantyne C.M., Soloviev D.A., Simon D.I., Plow E.F. (2008). Expression, activation, and function of integrin αMβ2 (Mac-1) on neutrophil-derived microparticles. Blood.

[37] Muralidharan-Chari V., Clancy J.W., Sedgwick A., D′Souza-Schorey C. (2010). Microvesicles: Mediators of extracellular communication during cancer progression. J. Cell Sci..

[38] Di Vizio D., Kim J., Hager M.H., Morello M., Yang W., Lafargue C.J., True L.D., Rubin M.A., Adam R.M., Beroukhim R. (2009). Oncosome formation in prostate cancer: Association with a region of frequent chromosomal deletion in metastatic disease. Cancer Res..

[39] Di Vizio D., Morello M., Dudley A.C., Schow P.W., Adam R.M., Morley S., Mulholland D., Rotinen M., Hager M.H., Insabato L. (2012). Large oncosomes in human prostate cancer tissues and in the circulation of mice with metastatic disease. Am. J. Pathol..

[40] Simpson R., Mathivanan S. (2012). Extracellular microvesicles: The need for internationally recognised nomenclature and stringent purification criteria. J. Proteom. Bioinform..

[41] Taylor R.C., Cullen S.P., Martin S.J. (2008). Apoptosis: Controlled demolition at the cellular level. Nat. Rev. Mol. Cell Biol..

[42] Kerr J.F., Wyllie A.H., Currie A.R. (1972). Apoptosis: A basic biological phenomenon with wide-ranging implications in tissue kinetics. Br. J. Cancer.

[43] Ciardiello C., Cavallini L., Spinelli C., Yang J., Reis-Sobreiro M., de Candia P., Minciacchi V.R., di Vizio D. (2016). Focus on extracellular vesicles: New frontiers of cell-to-cell communication in cancer. Int. J. Mol. Sci..

[44] Iraci N., Leonardi T., Gessler F., Vega B., Pluchino S. (2016). Focus on extracellular vesicles: Physiological role and signaling properties of extracellular membrane vesicles. Int. J. Mol. Sci..

[45] Ohno S.I., Drummen G.P.C., Kuroda M. (2016). Focus on extracellular vesicles: Development of exosome-based therapeutic systems. Int. J. Mol. Sci..

[46] Vella L.J., Hill A.F., Cheng L. (2016). Focus on extracellular vesicles: Exosomes and their role in protein trafficking in Alzheimer’s and Parkinson’s disease. Int. J. Mol. Sci..

[47] Zhang B., Tan K.H., Lim S.K. (2016). Focus on extracellular vesicles: Therapeutic efficacy of stem cell-derived extracellular vesicles. Int. J. Mol. Sci..

[48] Colombo M., Moita C., van Niel G., Kowal J., Vigneron J., Benaroch P., Manel N., Moita L.F., Thery C., Raposo G. (2013). Analysis of ESCRT functions in exosome biogenesis, composition and secretion highlights the heterogeneity of extracellular vesicles. J. Cell Sci..

[49] Pols M.S., Klumperman J. (2009). Trafficking and function of the tetraspanin CD63. Exp. Cell Res..

[50] Charrin S., le Naour F., Silvie O., Milhiet P.E., Boucheix C., Rubinstein E. (2009). Lateral organization of membrane proteins: Tetraspanins spin their web. Biochem. J..

[51] Chiaruttini N., Redondo-Morata L., Colom A., Humbert F., Lenz M., Scheuring S., Roux A. (2015). Relaxation of loaded ESCRT-III spiral springs drives membrane deformation. Cell.

[52] Raiborg C., Stenmark H. (2009). The ESCRT machinery in endosomal sorting of ubiquitylated membrane proteins. Nature.

[53] Hurley J.H. (2010). The ESCRT complexes. Crit. Rev. Biochem. Mol. Biol..

[54] Baietti M.F., Zhang Z., Mortier E., Melchior A., Degeest G., Geeraerts A., Ivarsson Y., Depoortere F., Coomans C., Vermeiren E. (2012). Syndecan-syntenin-ALIX regulates the biogenesis of exosomes. Nat. Cell Biol..

[55] Nabhan J.F., Hu R., Oh R.S., Cohen S.N., Lu Q. (2012). Formation and release of arrestin domain-containing protein 1-mediated microvesicles (ARMMs) at plasma membrane by recruitment of TSG101 protein. Proc. Natl. Acad. Sci. USA.

[56] Bissig C., Gruenberg J. (2014). ALIX and the multivesicular endosome: ALIX in Wonderland. Trends Cell Biol..

[57] Hurley J.H., Odorizzi G. (2012). Get on the exosome bus with ALIX. Nat. Cell Biol..

[58] Stuffers S., Sem Wegner C., Stenmark H., Brech A. (2009). Multivesicular endosome biogenesis in the absence of ESCRTs. Traffic.

[59] Trajkovic K., Hsu C., Chiantia S., Rajendran L., Wenzel D., Wieland F., Schwille P., Brügger B., Simons M. (2008). Ceramide triggers budding of exosome vesicles into multivesicular endosomes. Science.

[60] Wollert T., Hurley J.H. (2010). Molecular mechanism of multivesicular body biogenesis by ESCRT complexes. Nature.

[61] Ostrowski M., Carmo N.B., Krumeich S., Fanget I., Raposo G., Savina A., Moita C.F., Schauer K., Hume A.N., Freitas R.P. (2010). Rab27a and Rab27b control different steps of the exosome secretion pathway. Nat. Cell Biol..

[62] Savina A., Vidal M., Colombo M.I. (2002). The exosome pathway in K562 cells is regulated by Rab11. J. Cell Sci..

[63] Peinado H., Aleckovic M., Lavotshkin S., Matei I., Costa-Silva B., Moreno-Bueno G., Hergueta-Redondo M., Williams C., Garcia-Santos G., Ghajar C. (2012). Melanoma exosomes educate bone marrow progenitor cells toward a pro-metastatic phenotype through MET. Nat. Med..

[64] Miller K., Beardmore J., Kanety H., Schlessinger J., Hopkins C.R. (1986). Localization of the epidermal growth factor (EGF) receptor within the endosome of EGF-stimulated epidermoid carcinoma (A431) cells. J. Cell Biol..

[65] Sorkin A., Goh L.K. (2009). Endocytosis and intracellular trafficking of ErbBs. Exp. Cell Res..

[66] Bucci C., Thomsen P., Nicoziani P., McCarthy J., van Deurs B. (2000). Rab7: A key to lysosome biogenesis. Mol. Biol. Cell.

[67] Luzio J.P., Parkinson M.D., Gray S.R., Bright N.A. (2009). The delivery of endocytosed cargo to lysosomes. Biochem. Soc. Trans..

[68] Luzio J.P., Gray S.R., Bright N.A. (2010). Endosome-lysosome fusion. Biochem. Soc. Trans..

[69] Cocucci E., Racchetti G., Meldolesi J. (2009). Shedding microvesicles: Artefacts no more. Trends Cell Biol..

[70] Bevers E.M., Williamson P.L. (2010). Phospholipid scramblase: An update. FEBS Lett..

[71] Daleke D.L. (2003). Regulation of transbilayer plasma membrane phospholipid asymmetry. J. Lipid Res..

[72] Oram J.F., Vaughan A.M. (2000). ABCA1-mediated transport of cellular cholesterol and phospholipids to HDL apolipoproteins. Curr. Opin. Lipidol..

[73] Gonzalez L.J., Gibbons E., Bailey R.W., Fairbourn J., Nguyen T., Smith S.K., Best K.B., Nelson J., Judd A.M., Bell J.D. (2009). The influence of membrane physical properties on microvesicle release in human erythrocytes. PMC Biophys..

[74] Dekkers D.W., Comfurius P., Vuist W.M., Billheimer J.T., Dicker I., Weiss H.J., Zwaal R.F., Bevers E.M. (1998). Impaired Ca^2+^-induced tyrosine phosphorylation and defective lipid scrambling in erythrocytes from a patient with Scott syndrome: A study using an inhibitor for scramblase that mimics the defect in Scott syndrome. Blood.

[75] Suzuki J., Umeda M., Sims P.J., Nagata S. (2010). Calcium-dependent phospholipid scrambling by TMEM16F. Nature.

[76] Devaux P.F., Herrmann A., Ohlwein N., Kozlov M.M. (2008). How lipid flippases can modulate membrane structure. Biochim. Biophys. Acta.

[77] Elliott J.I., Sardini A., Cooper J.C., Alexander D.R., Davanture S., Chimini G., Higgins C.F. (2006). Phosphatidylserine exposure in B lymphocytes: A role for lipid packing. Blood.

[78] Jimenez J.J., Jy W., Mauro L.M., Soderland C., Horstman L.L., Ahn Y.S. (2003). Endothelial cells release phenotypically and quantitatively distinct microparticles in activation and apoptosis. Thromb. Res..

[79] Connor D.E., Exner T., Ma D.D., Joseph J.E. (2010). The majority of circulating platelet-derived microparticles fail to bind annexin V, lack phospholipid-dependent procoagulant activity and demonstrate greater expression of glycoprotein Ib. Thromb. Haemost..

[80] Rauch S., Martin-Serrano J. (2011). Multiple interactions between the ESCRT machinery and arrestin-related proteins: Implications for PPXY-dependent budding. J. Virol..

[81] Bianco F., Perrotta C., Novellino L., Francolini M., Riganti L., Menna E., Saglietti L., Schuchman E.H., Furlan R., Clementi E. (2009). Acid sphingomyelinase activity triggers microparticle release from glial cells. EMBO J..

[82] Curtis A.M., Wilkinson P.F., Gui M., Gales T.L., Hu E., Edelberg J.M. (2009). p38 mitogen-activated protein kinase targets the production of proinflammatory endothelial microparticles. J. Thromb. Haemost..

[83] Cocucci E., Racchetti G., Podini P., Meldolesi J. (2007). Enlargeosome traffic: Exocytosis triggered by various signals is followed by endocytosis, membrane shedding or both. Traffic.

[84] Baj-Krzyworzeka M., Szatanek R., Weglarczyk K., Baran J., Urbanowicz B., Branski P., Ratajczak M.Z., Zembala M. (2006). Tumour-derived microvesicles carry several surface determinants and mRNA of tumour cells and transfer some of these determinants to monocytes. Cancer Immunol. Immunother..

[85] Sidhu S.S., Mengistab A.T., Tauscher A.N., LaVail J., Basbaum C. (2004). The microvesicle as a vehicle for EMMPRIN in tumor-stromal interactions. Oncogene.

[86] Morel O., Toti F., Hugel B., Bakouboula B., Camoin-Jau L., Dignat-George F., Freyssinet J.M. (2006). Procoagulant microparticles: Disrupting the vascular homeostasis equation?. Arterioscler. Thromb. Vasc. Biol..

[87] Sinauridze E.I., Kireev D.A., Popenko N.Y., Pichugin A.V., Panteleev M.A., Krymskaya O.V., Ataullakhanov F.I. (2007). Platelet microparticle membranes have 50- to 100-fold higher specific procoagulant activity than activated platelets. Thromb. Haemost..

[88] Leroyer A.S., Tedgui A., Boulanger C.M. (2008). Role of microparticles in atherothrombosis. J. Intern. Med..

[89] Park S.Y., Jung M.Y., Kim H.J., Lee S.J., Kim S.Y., Lee B.H., Kwon T.H., Park R.W., Kim I.S. (2008). Rapid cell corpse clearance by stabilin-2, a membrane phosphatidylserine receptor. Cell Death Differ..

[90] DeKruyff R.H., Bu X., Ballesteros A., Santiago C., Chim Y.L., Lee H.H., Karisola P., Pichavant M., Kaplan G.G., Umetsu D.T. (2010). T cell/transmembrane, Ig, and mucin-3 allelic variants differentially recognize phosphatidylserine and mediate phagocytosis of apoptotic cells. J. Immunol..

[91] Hanayama R., Tanaka M., Miwa K., Shinohara A., Iwamatsu A., Nagata S. (2002). Identification of a factor that links apoptotic cells to phagocytes. Nature.

[92] Dale G.L. (2005). Coated-platelets: An emerging component of the procoagulant response. J. Thromb. Haemost..

[93] Nakano T., Ishimoto Y., Kishino J., Umeda M., Inoue K., Nagata K., Ohashi K., Mizuno K., Arita H. (1997). Cell adhesion to phosphatidylserine mediated by a product of growth arrest-specific gene 6. J. Biol. Chem..

[94] Lemke G., Rothlin C.V. (2008). Immunobiology of the TAM receptors. Nat. Rev. Immunol.

[95] Rezende S.M., Simmonds R.E., Lane D.A. (2004). Coagulation, inflammation, and apoptosis: Different roles for protein S and the protein S-C4b binding protein complex. Blood.

[96] Borisenko G.G., Matsura T., Liu S.X., Tyurin V.A., Jianfei J., Serinkan F.B., Kagan V.E. (2003). Macrophage recognition of externalized phosphatidylserine and phagocytosis of apoptotic Jurkat cells—Existence of a threshold. Arch. Biochem. Biophys..

[97] Gallucci S., Lolkema M., Matzinger P. (1999). Natural adjuvants: Endogenous activators of dendritic cells. Nat. Med..

[98] Kim H.S., Choi D.Y., Yun S.J., Choi S.M., Kang J.W., Jung J.W., Hwang D., Kim K.P., Kim D.W. (2012). Proteomic analysis of microvesicles derived from human mesenchymal stem cells. J. Proteome Res..

[99] Sadallah S., Eken C., Martin P.J., Schifferli J.A. (2011). Microparticles (ectosomes) shed by stored human platelets downregulate macrophages and modify the development of dendritic cells. J. Immunol..

[100] Azuma Y., Nakagawa H., Dote K., Higai K., Matsumoto K. (2011). Decreases in CD31 and CD47 levels on the cell surface during etoposide-induced Jurkat cell apoptosis. Biol. Pharm. Bull..

[101] Elmore S. (2007). Apoptosis: A review of programmed cell death. Toxicol. Pathol..

[102] Poon I.K., Lucas C.D., Rossi A.G., Ravichandran K.S. (2014). Apoptotic cell clearance: Basic biology and therapeutic potential. Nat. Rev. Immunol..

[103] Summers C., Rankin S.M., Condliffe A.M., Singh N., Peters A.M., Chilvers E.R. (2010). Neutrophil kinetics in health and disease. Trends Immunol..

[104] Witko-Sarsat V., Pederzoli-Ribeil M., Hirsch E., Sozzani S., Cassatella M.A. (2011). Regulating neutrophil apoptosis: New players enter the game. Trends Immunol..

[105] Pop C., Salvesen G.S. (2009). Human caspases: Activation, specificity, and regulation. J. Biol. Chem..

[106] Lane J.D., Allan V.J., Woodman P.G. (2005). Active relocation of chromatin and endoplasmic reticulum into blebs in late apoptotic cells. J. Cell Sci..

[107] Coleman M.L., Sahai E.A., Yeo M., Bosch M., Dewar A., Olson M.F. (2001). Membrane blebbing during apoptosis results from caspase-mediated activation of ROCK I. Nat. Cell Biol..

[108] Sebbagh M., Renvoize C., Hamelin J., Riche N., Bertoglio J., Breard J. (2001). Caspase-3-mediated cleavage of ROCK I induces MLC phosphorylation and apoptotic membrane blebbing. Nat. Cell Biol..

[109] Chang J., Xie M., Shah V.R., Schneider M.D., Entman M.L., Wei L., Schwartz R.J. (2006). Activation of Rho-associated coiled-coil protein kinase 1 (ROCK-1) by caspase-3 cleavage plays an essential role in cardiac myocyte apoptosis. Proc. Natl. Acad. Sci. USA.

[110] Kinchen J.M., Doukoumetzidis K., Almendinger J., Stergiou L., Tosello-Trampont A., Sifri C.D., Hengartner M.O., Ravichandran K.S. (2008). A pathway for phagosome maturation during engulfment of apoptotic cells. Nat. Cell Biol..

[111] Kinchen J.M., Ravichandran K.S. (2010). Identification of two evolutionarily conserved genes regulating processing of engulfed apoptotic cells. Nature.

[112] Suzuki J., Denning D.P., Imanishi E., Horvitz H.R., Nagata S. (2013). Xk-related protein 8 and CED-8 promote phosphatidylserine exposure in apoptotic cells. Science.

[113] Segawa K., Kurata S., Yanagihashi Y., Brummelkamp T.R., Matsuda F., Nagata S. (2014). Caspase-mediated cleavage of phospholipid flippase for apoptotic phosphatidylserine exposure. Science.

[114] Hagmann J., Burger M.M., Dagan D. (1999). Regulation of plasma membrane blebbing by the cytoskeleton. J. Cell. Biochem..

[115] Tinevez J.Y., Schulze U., Salbreux G., Roensch J., Joanny J.F., Paluch E. (2009). Role of cortical tension in bleb growth. Proc. Natl. Acad. Sci. USA.

[116] Kothakota S., Azuma T., Reinhard C., Klippel A., Tang J., Chu K., McGarry T.J., Kirschner M.W., Koths K., Kwiatkowski D.J. (1997). Caspase-3-generated fragment of gelsolin: Effector of morphological change in apoptosis. Science.

[117] Witasp E., Uthaisang W., Elenstrom-Magnusson C., Hanayama R., Tanaka M., Nagata S., Orrenius S., Fadeel B. (2007). Bridge over troubled water: Milk fat globule epidermal growth factor 8 promotes human monocyte-derived macrophage clearance of non-blebbing phosphatidylserine-positive target cells. Cell Death Differ..

[118] Barry D.J., Durkin C.H., Abella J.V., Way M. (2015). Open source software for quantification of cell migration, protrusions, and fluorescence intensities. J. Cell Biol..

[119] Charras G.T., Hu C.K., Coughlin M., Mitchison T.J. (2006). Reassembly of contractile actin cortex in cell blebs. J. Cell Biol..

[120] Moss D.K., Betin V.M., Malesinski S.D., Lane J.D. (2006). A novel role for microtubules in apoptotic chromatin dynamics and cellular fragmentation. J. Cell Sci..

[121] Poon I.K., Chiu Y.H., Armstrong A.J., Kinchen J.M., Juncadella I.J., Bayliss D.A., Ravichandran K.S. (2014). Unexpected link between an antibiotic, pannexin channels and apoptosis. Nature.

[122] Chekeni F.B., Elliott M.R., Sandilos J.K., Walk S.F., Kinchen J.M., Lazarowski E.R., Armstrong A.J., Penuela S., Laird D.W., Salvesen G.S. (2010). Pannexin 1 channels mediate “find-me” signal release and membrane permeability during apoptosis. Nature.

[123] Atkin-Smith G.K., Tixeira R., Paone S., Mathivanan S., Collins C., Liem M., Goodall K.J., Ravichandran K.S., Hulett M.D., Poon I.K. (2015). A novel mechanism of generating extracellular vesicles during apoptosis via a beads-on-a-string membrane structure. Nat. Commun..

[124] Théry C., Ostrowski M., Segura E. (2009). Membrane vesicles as conveyors of immune responses. Nat. Rev. Immunol..

[125] Carroll-Portillo A., Surviladze Z., Cambi A., Lidke D.S., Wilson B.S. (2012). Mast cell synapses and exosomes: Membrane contacts for information exchange. Front. Immunol..

[126] Lydic T.A., Townsend S., Adda C.G., Collins C., Mathivanan S., Reid G.E. (2015). Rapid and comprehensive “shotgun” lipidome profiling of colorectal cancer cell derived exosomes. Methods.

[127] Simpson R.J., Kalra H., Mathivanan S. (2012). ExoCarta as a resource for exosomal research. J. Extracell. Vesicles.

[128] Mathivanan S., Fahner C.J., Reid G.E., Simpson R.J. (2012). ExoCarta 2012: Database of exosomal proteins, RNA and lipids. Nucleic Acids Res..

[129] Mathivanan S., Simpson R.J. (2009). ExoCarta: A compendium of exosomal proteins and RNA. Proteomics.

[130] Kalra H., Simpson R.J., Ji H., Aikawa E., Altevogt P., Askenase P., Bond V.C., Borràs F.E., Breakefield X., Budnik V. (2012). Vesiclepedia: A compendium for extracellular vesicles with continuous community annotation. PLoS Biol..

[131] Conde-Vancells J., Rodriguez-Suarez E., Embade N., Gil D., Matthiesen R., Valle M., Elortza F., Lu S.C., Mato J.M., Falcon-Perez J.M. (2008). Characterization and comprehensive proteome profiling of exosomes secreted by hepatocytes. J. Proteome Res..

[132] Subra C., Grand D., Laulagnier K., Stella A., Lambeau G., Paillasse M., de Medina P., Monsarrat B., Perret B., Silvente-Poirot S. (2010). Exosomes account for vesicle-mediated transcellular transport of activatable phospholipases and prostaglandins. J. Lipid Res..

[133] Mathivanan S., Lim J.W., Tauro B.J., Ji H., Moritz R.L., Simpson R.J. (2010). Proteomics analysis of A33 immunoaffinity-purified exosomes released from the human colon tumor cell line LIM1215 reveals a tissue-specific protein signature. Mol. Cell. Proteom..

[134] Subra C., Laulagnier K., Perret B., Record M. (2007). Exosome lipidomics unravels lipid sorting at the level of multivesicular bodies. Biochimie.

[135] Wubbolts R., Leckie R.S., Veenhuizen P.T., Schwarzmann G., Möbius W., Hoernschemeyer J., Slot J.-W., Geuze H.J., Stoorvogel W. (2003). Proteomic and biochemical analyses of human B cell-derived exosomes: Potential implications for their function and multivesicular body formation. J. Biol. Chem..

[136] Brouwers J.F., Aalberts M., Jansen J.W., van Niel G., Wauben M.H., Stout T.A., Helms J.B., Stoorvogel W. (2013). Distinct lipid compositions of two types of human prostasomes. Proteomics.

[137] Laulagnier K., Motta C., Hamdi S., Roy S., Fauvelle F., Pageaux J.F., Kobayashi T., Salles J.P., Perret B., Bonnerot C. (2004). Mast cell- and dendritic cell-derived exosomes display a specific lipid composition and an unusual membrane organization. Biochem. J..

[138] Matsuo H., Chevallier J., Mayran N., le Blanc I., Ferguson C., Faure J., Blanc N.S., Matile S., Dubochet J., Sadoul R. (2004). Role of LBPA and Alix in multivesicular liposome formation and endosome organization. Science.

[139] Batista B.S., Eng W.S., Pilobello K.T., Hendricks-Muñoz K.D., Mahal L.K. (2011). Identification of a conserved glycan signature for microvesicles. J. Proteome Res..

[140] Saunderson S.C., Dunn A.C., Crocker P.R., McLellan A.D. (2014). CD169 mediates the capture of exosomes in spleen and lymph node. Blood.

[141] Al-Nedawi K., Meehan B., Micallef J., Lhotak V., May L., Guha A., Rak J. (2008). Intercellular transfer of the oncogenic receptor EGFRvIII by microvesicles derived from tumour cells. Nat. Cell Biol..

[142] Bernimoulin M., Waters E.K., Foy M., Steele B.M., Sullivan M., Falet H., Walsh M.T., Barteneva N., Geng J.G., Hartwig J.H. (2009). Differential stimulation of monocytic cells results in distinct populations of microparticles. J. Thromb. Haemost..

[143] Lunavat T.R., Cheng L., Kim D.K., Bhadury J., Jang S.C., Lasser C., Sharples R.A., Lopez M.D., Nilsson J., Gho Y.S. (2015). Small RNA deep sequencing discriminates subsets of extracellular vesicles released by melanoma cells—Evidence of unique microRNA cargos. RNA Biol..

[144] Weerheim A.M., Kolb A.M., Sturk A., Nieuwland R. (2002). Phospholipid composition of cell-derived microparticles determined by one-dimensional high-performance thin-layer chromatography. Anal. Biochem..

[145] Losito I., Patruno R., Conte E., Cataldi T.R., Megli F.M., Palmisano F. (2013). Phospholipidomics of human blood microparticles. Anal. Chem..

[146] Mallat Z., Hugel B., Ohan J., Leseche G., Freyssinet J.M., Tedgui A. (1999). Shed membrane microparticles with procoagulant potential in human atherosclerotic plaques: A role for apoptosis in plaque thrombogenicity. Circulation.

[147] Turiak L., Misjak P., Szabo T.G., Aradi B., Paloczi K., Ozohanics O., Drahos L., Kittel A., Falus A., Buzas E.I. (2011). Proteomic characterization of thymocyte-derived microvesicles and apoptotic bodies in BALB/c mice. J. Proteom..

[148] Lleo A., Zhang W., McDonald W.H., Seeley E.H., Leung P.S., Coppel R.L., Ansari A.A., Adams D.H., Afford S., Invernizzi P. (2014). Shotgun proteomics: Identification of unique protein profiles of apoptotic bodies from biliary epithelial cells. Hepatology.

[149] Choi D.S., Kim D.K., Kim Y.K., Gho Y.S. (2013). Proteomics, transcriptomics and lipidomics of exosomes and ectosomes. Proteomics.

[150] Oh S., Kang D., Ahn S.M., Simpson R.J., Lee B.H., Moon M.H. (2007). Miniaturized asymmetrical flow field-flow fractionation: Application to biological vesicles. J. Sep. Sci..

[151] Taylor D.D., Shah S. (2015). Methods of isolating extracellular vesicles impact down-stream analyses of their cargoes. Methods.

[152] Momen-Heravi F., Balaj L., Alian S., Mantel P.-Y., Halleck A.E., Trachtenberg A.J., Soria C.E., Oquin S., Bonebreak C.M., Saracoglu E. (2013). Current methods for the isolation of extracellular vesicles. Biol. Chem..

[153] Sáenz-Cuesta M., Arbelaiz A., Oregi A., Irizar H., Osorio-Querejeta I., Muñoz-Culla M., Banales J.M., Falcón-Pérez J.M., Olascoaga J., Otaegui D. (2015). Methods for extracellular vesicles isolation in a hospital setting. Front. Immunol..

[154] Tauro B.J., Greening D.W., Mathias R.A., Ji H., Mathivanan S., Scott A.M., Simpson R.J. (2012). Comparison of ultracentrifugation, density gradient separation, and immunoaffinity capture methods for isolating human colon cancer cell line LIM1863-derived exosomes. Methods.

[155] Cheruvanky A., Zhou H., Pisitkun T., Kopp J.B., Knepper M.A., Yuen P.S., Star R.A. (2007). Rapid isolation of urinary exosomal biomarkers using a nanomembrane ultrafiltration concentrator. Am. J. Physiol. Ren. Physiol..

[156] Merchant M.L., Powell D.W., Wilkey D.W., Cummins T.D., Deegens J.K., Rood I.M., McAfee K.J., Fleischer C., Klein E., Klein J.B. (2010). Microfiltration isolation of human urinary exosomes for characterization by MS. Proteom. Clin. Appl..

[157] Boukouris S., Mathivanan S. (2015). Exosomes in bodily fluids are a highly stable resource of disease biomarkers. Proteom. Clin. Appl..

[158] Lázaro-Ibáñez E., Sanz-Garcia A., Visakorpi T., Escobedo-Lucea C., Siljander P., Ayuso-Sacido A., Yliperttula M. (2014). Different gDNA content in the subpopulations of prostate cancer extracellular vesicles: Apoptotic bodies, microvesicles, and exosomes. Prostate.

[159] Hristov M., Erl W., Linder S., Weber P.C. (2004). Apoptotic bodies from endothelial cells enhance the number and initiate the differentiation of human endothelial progenitor cells *in vitro*. Blood.

[160] Cantin R., Diou J., Bélanger D., Tremblay A.M., Gilbert C. (2008). Discrimination between exosomes and HIV-1: Purification of both vesicles from cell-free supernatants. J. Immunol. Methods.

[161] Gyorgy B., Modos K., Pallinger E., Paloczi K., Pasztoi M., Misjak P., Deli M., Sipos A., Szalai A., Voszka I. (2011). Detection and isolation of cell-derived microparticles are compromised by protein complexes due to shared biophysical parameters. Blood.

[162] Rood I.M., Deegens J.K., Merchant M.L., Tamboer W.P., Wilkey D.W., Wetzels J.F., Klein J.B. (2010). Comparison of three methods for isolation of urinary microvesicles to identify biomarkers of nephrotic syndrome. Kidney Int..

[163] Webber J., Clayton A. (2013). How pure are your vesicles?. J. Extracell. Vesicles.

[164] Nordin J.Z., Lee Y., Vader P., Mager I., Johansson H.J., Heusermann W., Wiklander O.P., Hallbrink M., Seow Y., Bultema J.J. (2015). Ultrafiltration with size-exclusion liquid chromatography for high yield isolation of extracellular vesicles preserving intact biophysical and functional properties. Nanomed.: Nanotechnol. Biol. Med..

[165] Logozzi M., de Milito A., Lugini L., Borghi M., Calabro L., Spada M., Perdicchio M., Marino M.L., Federici C., Iessi E. (2009). High levels of exosomes expressing CD63 and caveolin-1 in plasma of melanoma patients. PLoS ONE.

[166] Qu J.L., Qu X.J., Zhao M.F., Teng Y.E., Zhang Y., Hou K.Z., Jiang Y.H., Yang X.H., Liu Y.P. (2009). The role of cbl family of ubiquitin ligases in gastric cancer exosome-induced apoptosis of Jurkat T cells. Acta Oncol..

[167] Ochieng J., Pratap S., Khatua A.K., Sakwe A.M. (2009). Anchorage-independent growth of breast carcinoma cells is mediated by serum exosomes. Exp. Cell Res..

[168] McCready J., Sims J.D., Chan D., Jay D.G. (2010). Secretion of extracellular hsp90α via exosomes increases cancer cell motility: A role for plasminogen activation. BMC Cancer.

[169] Jung T., Castellana D., Klingbeil P., Cuesta Hernandez I., Vitacolonna M., Orlicky D.J., Roffler S.R., Brodt P., Zoller M. (2009). CD44v6 dependence of premetastatic niche preparation by exosomes. Neoplasia.

[170] Skog J., Wurdinger T., van Rijn S., Meijer D.H., Gainche L., Sena-Esteves M., Curry W.T. Jr., Carter B.S., Krichevsky A.M., Breakefield X.O. (2008). Glioblastoma microvesicles transport RNA and proteins that promote tumour growth and provide diagnostic biomarkers. Nat. Cell Biol..

[171] Ristorcelli E., Beraud E., Verrando P., Villard C., Lafitte D., Sbarra V., Lombardo D., Verine A. (2008). Human tumor nanoparticles induce apoptosis of pancreatic cancer cells. FASEB J..

[172] Zhang Y., Luo C.L., He B.C., Zhang J.M., Cheng G., Wu X.H. (2010). Exosomes derived from IL-12-anchored renal cancer cells increase induction of specific antitumor response *in vitro*: A novel vaccine for renal cell carcinoma. Int. J. Oncol..

[173] Sharples R.A., Vella L.J., Nisbet R.M., Naylor R., Perez K., Barnham K.J., Masters C.L., Hill A.F. (2008). Inhibition of gamma-secretase causes increased secretion of amyloid precursor protein C-terminal fragments in association with exosomes. FASEB J..

[174] Perez-Gonzalez R., Gauthier S.A., Kumar A., Levy E. (2012). The exosome secretory pathway transports amyloid precursor protein carboxyl-terminal fragments from the cell into the brain extracellular space. J. Biol. Chem..

[175] Yuyama K., Sun H., Sakai S., Mitsutake S., Okada M., Tahara H., Furukawa J., Fujitani N., Shinohara Y., Igarashi Y. (2014). Decreased amyloid-beta pathologies by intracerebral loading of glycosphingolipid-enriched exosomes in Alzheimer model mice. J. Biol. Chem..

[176] Emmanouilidou E., Melachroinou K., Roumeliotis T., Garbis S.D., Ntzouni M., Margaritis L.H., Stefanis L., Vekrellis K. (2010). Cell-produced alpha-synuclein is secreted in a calcium-dependent manner by exosomes and impacts neuronal survival. J. Neurosci..

[177] Tsunemi T., Hamada K., Krainc D. (2014). ATP13A2/PARK9 regulates secretion of exosomes and alpha-synuclein. J. Neurosci..

[178] Shi M., Liu C., Cook T.J., Bullock K.M., Zhao Y., Ginghina C., Li Y., Aro P., Dator R., He C. (2014). Plasma exosomal alpha-synuclein is likely CNS-derived and increased in Parkinson′s disease. Acta Neuropathol..

[179] Ratajczak J., Wysoczynski M., Hayek F., Janowska-Wieczorek A., Ratajczak M.Z. (2006). Membrane-derived microvesicles: Important and underappreciated mediators of cell-to-cell communication. Leukemia.

[180] Gu S., Zhang W., Chen J., Ma R., Xiao X., Ma X., Yao Z., Chen Y. (2014). Epc-derived microvesicles protect cardiomyocytes from Ang II-induced hypertrophy and apoptosis. PLoS ONE.

[181] Lai R.C., Arslan F., Lee M.M., Sze N.S., Choo A., Chen T.S., Salto-Tellez M., Timmers L., Lee C.N., el Oakley R.M. (2010). Exosome secreted by MSC reduces myocardial ischemia/reperfusion injury. Stem Cell. Res..

[182] Kogianni G., Mann V., Noble B.S. (2008). Apoptotic bodies convey activity capable of initiating osteoclastogenesis and localized bone destruction. J. Bone Miner. Res..

[183] Jiang J.X., Mikami K., Venugopal S., Li Y., Torok N.J. (2009). Apoptotic body engulfment by hepatic stellate cells promotes their survival by the JAK/STAT and Akt/NF-κB-dependent pathways. J. Hepatol..

[184] Marin-Gallen S., Clemente-Casares X., Planas R., Pujol-Autonell I., Carrascal J., Carrillo J., Ampudia R., Verdaguer J., Pujol-Borrell R., Borras F.E. (2010). Dendritic cells pulsed with antigen-specific apoptotic bodies prevent experimental type 1 diabetes. Clin. Exp. Immunol..

[185] Schiller M., Bekeredjian-Ding I., Heyder P., Blank N., Ho A.D., Lorenz H.M. (2008). Autoantigens are translocated into small apoptotic bodies during early stages of apoptosis. Cell. Death Differ..

[186] Raposo G., Nijman H.W., Stoorvogel W., Liejendekker R., Harding C.V., Melief C., Geuze H.J. (1996). B lymphocytes secrete antigen-presenting vesicles. J. Exp. Med..

[187] Admyre C., Johansson S.M., Paulie S., Gabrielsson S. (2006). Direct exosome stimulation of peripheral human T cells detected by ELISPOT. Eur. J. Immunol..

[188] Montecalvo A., Shufesky W.J., Stolz D.B., Sullivan M.G., Wang Z., Divito S.J., Papworth G.D., Watkins S.C., Robbins P.D., Larregina A.T. (2008). Exosomes as a short-range mechanism to spread alloantigen between dendritic cells during T cell allorecognition. J. Immunol..

[189] Mallegol J., van Niel G., Lebreton C., Lepelletier Y., Candalh C., Dugave C., Heath J.K., Raposo G., Cerf-Bensussan N., Heyman M. (2007). T84-intestinal epithelial exosomes bear MHC class II/peptide complexes potentiating antigen presentation by dendritic cells. Gastroenterology.

[190] Segura E., Guerin C., Hogg N., Amigorena S., Thery C. (2007). CD8+ dendritic cells use LFA-1 to capture MHC-peptide complexes from exosomes *in vivo*. J. Immunol..

[191] Théry C., Duban L., Segura E., Veron P., Lantz O., Amigorena S. (2002). Indirect activation of naive CD4+ T cells by dendritic cell-derived exosomes. Nat. Immunol..

[192] Walker J.D., Maier C.L., Pober J.S. (2009). Cytomegalovirus-infected human endothelial cells can stimulate allogeneic CD4+ memory T cells by releasing antigenic exosomes. J. Immunol..

[193] Giri P.K., Schorey J.S. (2008). Exosomes derived from M. Bovis BCG infected macrophages activate antigen-specific CD4+ and CD8+ T cells *in vitro* and *in vivo*. PLoS ONE.

[194] Papp K., Vegh P., Prechl J., Kerekes K., Kovacs J., Csikos G., Bajtay Z., Erdei A. (2008). B lymphocytes and macrophages release cell membrane deposited C3-fragments on exosomes with T cell response-enhancing capacity. Mol. Immunol..

[195] Muntasell A., Berger A.C., Roche P.A. (2007). T cell-induced secretion of MHC class II-peptide complexes on B cell exosomes. EMBO J..

[196] Ostman S., Taube M., Telemo E. (2005). Tolerosome-induced oral tolerance is MHC dependent. Immunology.

[197] Skokos D., Botros H.G., Demeure C., Morin J., Peronet R., Birkenmeier G., Boudaly S., Mecheri S. (2003). Mast cell-derived exosomes induce phenotypic and functional maturation of dendritic cells and elicit specific immune responses *in vivo*. J. Immunol..

[198] Robbins P.D., Morelli A.E. (2014). Regulation of immune responses by extracellular vesicles. Nat. Rev. Immunol..

[199] Mitchell P., Petfalski E., Shevchenko A., Mann M., Tollervey D. (1997). The exosome: A conserved eukaryotic RNA processing complex containing multiple 3′→5′ exoribonucleases. Cell.

[200] Takahashi Y., Nishikawa M., Shinotsuka H., Matsui Y., Ohara S., Imai T., Takakura Y. (2013). Visualization and *in vivo* tracking of the exosomes of murine melanoma B16-BL6 cells in mice after intravenous injection. J. Biotechnol..

[201] Morishita M., Takahashi Y., Nishikawa M., Sano K., Kato K., Yamashita T., Imai T., Saji H., Takakura Y. (2015). Quantitative analysis of tissue distribution of the B16BL6-derived exosomes using a streptavidin-lactadherin fusion protein and iodine-125-labeled biotin derivative after intravenous injection in mice. J. Pharm. Sci..

[202] Willekens F.L., Werre J.M., Kruijt J.K., Roerdinkholder-Stoelwinder B., Groenen-Dopp Y.A., van den Bos A.G., Bosman G.J., van Berkel T.J. (2005). Liver Kupffer cells rapidly remove red blood cell-derived vesicles from the circulation by scavenger receptors. Blood.

